# Exploring primary school years interactions around child weight: A qualitative meta‐synthesis of school staff, parent, and child views and experiences

**DOI:** 10.1111/obr.13451

**Published:** 2022-04-10

**Authors:** Anna Chisholm, Nia Coupe, Katalin Ujhelyi Gomez, Jo Hart, Sarah Peters

**Affiliations:** ^1^ Department of Psychology University of Liverpool Liverpool UK; ^2^ Division of Medical Education University of Manchester Manchester UK; ^3^ Division of Psychology and Mental Health University of Manchester Manchester UK

**Keywords:** child weight, interaction, qualitative, school

## Abstract

Interactions about children's weight and weight‐related behaviors occur from an early stage in school settings between various stakeholders and are often intended to facilitate weight‐related behavioral change in children and/or families. This meta‐synthesis (PROSPERO ‐ CRD42019133231) aimed to investigate stakeholder reported experiences and challenges of these encounters. Studies were eligible if they included school stakeholders (teaching or nonteaching staff, parents, caregivers, or children), explored communication topics related to child obesity (weight, diet or activity), were conducted within an early school setting (primary school stage or international equivalent), and used qualitative methods. Database searches conducted March–July 2019 (updated November 2020) identified 40 studies (2324 participants) from seven countries. Included studies were assessed for quality using the Critical Appraisal Skills Programme. Using inductive thematic analysis, we identified four core themes across this database: (1) “conversation characteristics and consequences,” (2) “missing components,” (3) “avoiding stigma,” and (4) “school responsibilities.” Overall, stakeholders recognized that schools are well‐positioned to provide positive influential messages about childhood obesity and reported that discussions on this topic do occur in early school settings but that stakeholders find them difficult, complex, and lack the necessary skills to deliver the nonjudgmental, consistent, and tailored support that they desire.

## 1 INTRODUCTION

Obesity is one of the leading risk factors for current mortality and morbidity including cardiovascular disease, diabetes, and musculoskeletal diseases.^1^ Obesity has now overtaken smoking as the leading cause of several cancers including bowel, kidney, ovary, and liver cancer and is the second greatest attributable risk factor for cancer overall.^2^ Across the world, obesity therefore remains a major threat to public health. As with adults, childhood obesity is now recognized as a global epidemic, being caused and maintained by complex interactions between genetic, behavioral, social, and environmental factors.^3^ The nature of these determinants indicates that obesity is not a result of a lack of willpower or down to individual choice, and despite this, it is preventable.^4^ It is recognized that obesity interventions should occur from an early age to prevent potential consequences on individual's physical health and well‐being, and thus, efforts to reduce childhood obesity are widespread, targeting family, hospital, community, and school settings.^3^ However, childhood obesity continues to rise year on year with 14.4 million children now affected in the United States (USA) (19.3% of 2–19 years old),^5^ and in the United Kingdom (UK), latest observations show that obesity more than doubles from the first (age 4/5) to the last year (age 10/11) of primary school (9.9% to 21%, respectively, in 2019–2020).^6^

School health promotion including encouraging healthy eating and physical activity has been previously characterized as fragmented, low priority, and uncoordinated,^7^ leading to the WHO introducing the *Health Promoting Schools* initiative in 1996, encouraging structural changes to schools' physical and social environments.^8^ Following this, many primary school‐based interventions addressing child obesity have focused on making modifications to elements such as school playgrounds, break time activity opportunities, and school lunch options.^7^, ^9^ Successful implementation of these interventions has led to a range of positive outcomes likely to contribute towards tackling child obesity such as changing in‐school food consumption, increasing child activity levels, and facilitating wider school‐based health promotion activities.^7^, ^10^, ^11^ However, school‐based interventions and outcomes often target in‐school behavior and may not extend beyond this or consider how best to support parents, children, and families with lasting changes made outside of school. Although parents and children have reported making behavioral changes following improvements to schools' healthy living curricula, further research is required to identify its influence on home‐based behavior.^12^

Regarding the role of primary schools in preventing and controlling child obesity, national guidelines emphasize that schools' main strategy for intervention should align with their greatest strength—effective education (and not go too far beyond this), but they also clearly identify a role for primary schools in providing consistent messages to children and families that reinforce positive health‐related behavior in the context of child obesity.^12^ While international guidance frequently prioritizes addressing child obesity via optimizing the school food and activity environment, the curricula, and monitoring child weight,^13^, ^14^ some guidelines outline additional areas for improvement such as individualized staff‐parent or staff‐child discussion around healthy eating and activity (e.g., greater need to listen to parents regarding home‐life complexities, providing parents with feedback about children's physical activity, and advising on what could be done at home to build upon healthy living learning at school).^12^ Facilitating pathways for referral to health professionals such as school nurses is also highlighted as a key role for schools, although limited resource and access to these services have been recognized as significant barriers in the United Kingdom.^12^ In the United States, national guidelines emphasize that school‐based obesity interventions should incorporate a range of stakeholders (e.g., parents, children, and communities) and services (e.g., health, mental health, and social services). There is therefore potential for wide ranging but not necessarily clearly articulated roles for school staff in this area.

Regardless of the specific roles for school staff in addressing child obesity, evidence suggests that parental and family involvement in school‐based interventions is helpful in increasing the chances of directly preventing and controlling child obesity.^16^, ^17^ This suggests that staff–parent relationships and inevitable discussions between these parties are likely to be of importance. Opportunistic child weight discussions are likely to arise between staff and children, for example, when delivering healthy living curricula or during lunchtimes or activity breaks or in response to adverse events like withdrawal from PE classes or weight‐related bullying.^18^ However, it is well‐known that discussions involving health‐related behavior (including changes to diet, activity, and obesity management) can be experienced by those involved as highly sensitive and difficult to raise or to engage in.^19^ In school settings, head teachers have reported that the most significant barrier to effective action on obesity prevention is the lack of knowledge, awareness, and skills to deal with the complexities and sensitivities of child obesity.^20^ Alternatively, school nurses report being well placed to address obesity with children in schools and report viewing their role as providing emotional support to children and working with children and their parents but can also find these conversations highly challenging, for example, due to lacking knowledge of complex motivational skills necessary for effective conversations.^21^, ^22^ Together, this suggests that there are a range of unaddressed challenges that primary school staff currently face in relation to child obesity management depending on discipline and position within the school setting. Obtaining a fuller understanding of the barriers and facilitators to child weight discussions within primary schools would therefore be beneficial in terms of (1) identifying existing influences on child weight discussions and (2) informing future strategies to optimize these discussions.

A recent review identified barriers to effective child weight discussions that occur within health care settings, including lack of staff knowledge and skill to manage the complexities of child obesity, concern for providing inconsistent advice, a sense of futility in engaging in weight management conversations, and fears around damaging the professional–parent relationship.^23^ Barriers related to parental factors included a perceived lack of family motivation and inability to recognize obesity in children, while organizational factors included limited time, resources, support, contact opportunities, and referral protocols.^23^ Given that there is overlap between the professionals in this sample (e.g., nurses, doctors, dieticians, psychologists, and clinical managers) and those working in primary schools (e.g., school nurses), it is possible that some of these findings are transferable to primary school settings. However, without consolidation of the evidence specific to child weight discussions, the key challenges faced by individuals in this setting remain unknown. Finally, given the combined emphasis on rapidly rising obesity rates in early school years settings and calls to prevent and manage childhood from an early age, we chose to focus on primary school level interactions in this study. We therefore conducted a systematic review of the experiences and views of those involved in primary school‐based child weight discussions (referred to from here onwards as “stakeholders”) in line with an intervention mapping approach to health intervention designs in which a detailed description of the problem as associated challenges is specified, by drawing on existing research findings to help explore unanswered questions.^24^

Central research question: What are primary school stakeholders' views and experiences of child weight conversations in school settings?

Subquestions: (1) What are the barriers and facilitators to effective child weight discussions as experienced by primary school stakeholders? (2) What role do primary school stakeholders have in the context of addressing child weight?

## 2 METHODS

We conducted a systematic review and meta‐synthesis^25^ of qualitative studies looking at stakeholder views and experiences of child weight conversations within primary school settings. An inductive thematic analysis approach was taken as the aim was to explore stakeholder views and experience and synthesize existing qualitative research findings to identify patterns across the existing qualitative dataset without predetermining the structure of the results. The study protocol is available on the PROSPERO register (CRD42019133231), and this report follows ENTREQ guidance for reporting qualitative syntheses^26^ and PRISMA guidelines.^27^ Supporting information File S1 displays completed guideline checklists in full. Four databases were searched between March 25, 2019 and July 15, 2019 and updated in November 2020. Database selection was based on a previous similar review^23^ including Medline, CINHAL, and PsychInfo, with the addition of Research Education Complete Database given the focus of this review on school settings. Search terms related to stakeholder groups, communication topics, school settings, and qualitative study designs. Database specific “MeSH” terms or subject headings were used along with Boolean operators (AND/OR) to combine categories of search terms and to adapt the search strategy to each database (supporting information File S2 shows full search strategy).

### 2.1 Eligibility

Studies were eligible if they explored stakeholders' views or experiences of child weight‐related conversations in primary school settings. Stakeholders were defined as school staff including teaching, nonteaching staff or school health professionals, parents, carers, or children. Studies were included if (1) participants included at least one stakeholder group, (2) the setting was an early school setting (defined as a primary school, elementary school or other international equivalent), (3) if data are related to the topic of child weight communication (including written or verbal interactions about child weight, diet or physical activity), and (4) if the study used a qualitative research design (including mixed methods designs or qualitative data collection embedded within larger trials). Where studies included more than one population or study topic, only data relevant to the research questions were extracted and included in the meta‐synthesis. On occasions where the data source was ambiguous (e.g., data from primary and secondary school students were collated and not separately identified), we chose to be inclusive and retain relevant data within the synthesis. No geographical or time restrictions were placed on searches, though searches were limited to retrieving English language texts. Full inclusion and exclusion criteria are displayed in Table 1. The final search strategy was piloted and refined via initial scoping searches, and in addition to running database searches, we ran “similar articles” searches on databases and conducted manual searches of reference lists for relevant reviews and articles.

**TABLE 1:** Full eligibility criteria

Study component	Inclusion criteria	Exclusion criteria
Setting	Schools defined as Primary School Level or international equivalents of this school level across any country, that is, Elementary or Infant School Level. Can be defined in terms of level of the school or stage of school, that is, grade or year. Include reception to Year 6 (UK system: 5–11 years old) Include kindergarten (K)—Grade 5 (US system: 4–11 years old) Include Senior Kindergarten—Grade 5 (Canadian system: 5–11 years old) For further international comparisons see guides: http://www.free‐for‐kids.com/uk‐us‐education‐systems.shtml https://www.ourkids.net/school/canada‐grade‐levels	Preschool, kindergarten for 0–4 year olds, middle school (11 years old and over), secondary school, high school, college, university
Population	Primary school stakeholders including the following groups: General or specialist educators, teachers, teaching assistants, head/principle teacher, or school governors, School nurses, school dieticians/nutritionists, or nonteaching staff that work within primary school settings, Parents, guardians, or family members of children attending primary school, Students/children that attend primary school	Preschool children (0–4 years old) Children in middle school or secondary school or beyond (including all international equivalents; see above) Staff that only work outside primary school settings Parents that do not have children currently attending primary school (or international equivalent school level)
Study topic	Collected study data must refer to communication, interaction, or talk between stakeholders. Interactions must relate to child weight, growth, body mass index, diet, or physical activity (can include conversations about diet or activity that do not specifically refer to weight) Information exchange or interactions can occur via electronic, web, or written formats as long as interaction is between primary school stake holder groups	Information provision that is didactic in nature and part of existing school curriculum (e.g., school lesson on healthy eating without discussion fostered between stakeholder groups) Study seeks only to test or assess stakeholder knowledge of child weight, BMI, growth, diet, or activity.
Study design	Qualitative study design: Qualitative design is primary focus of the study (e.g., interview or focus group study) Qualitative data are part of mixed‐method or multimethod design (e.g., questionnaire study including open ended questions that are analyzed qualitatively) Qualitative study embedded within larger study design (e.g., process evaluation of an RCT)	Quantitative study only (no qualitative analysis of data included in study)

### 2.2 Screening

**FIGURE 1:**
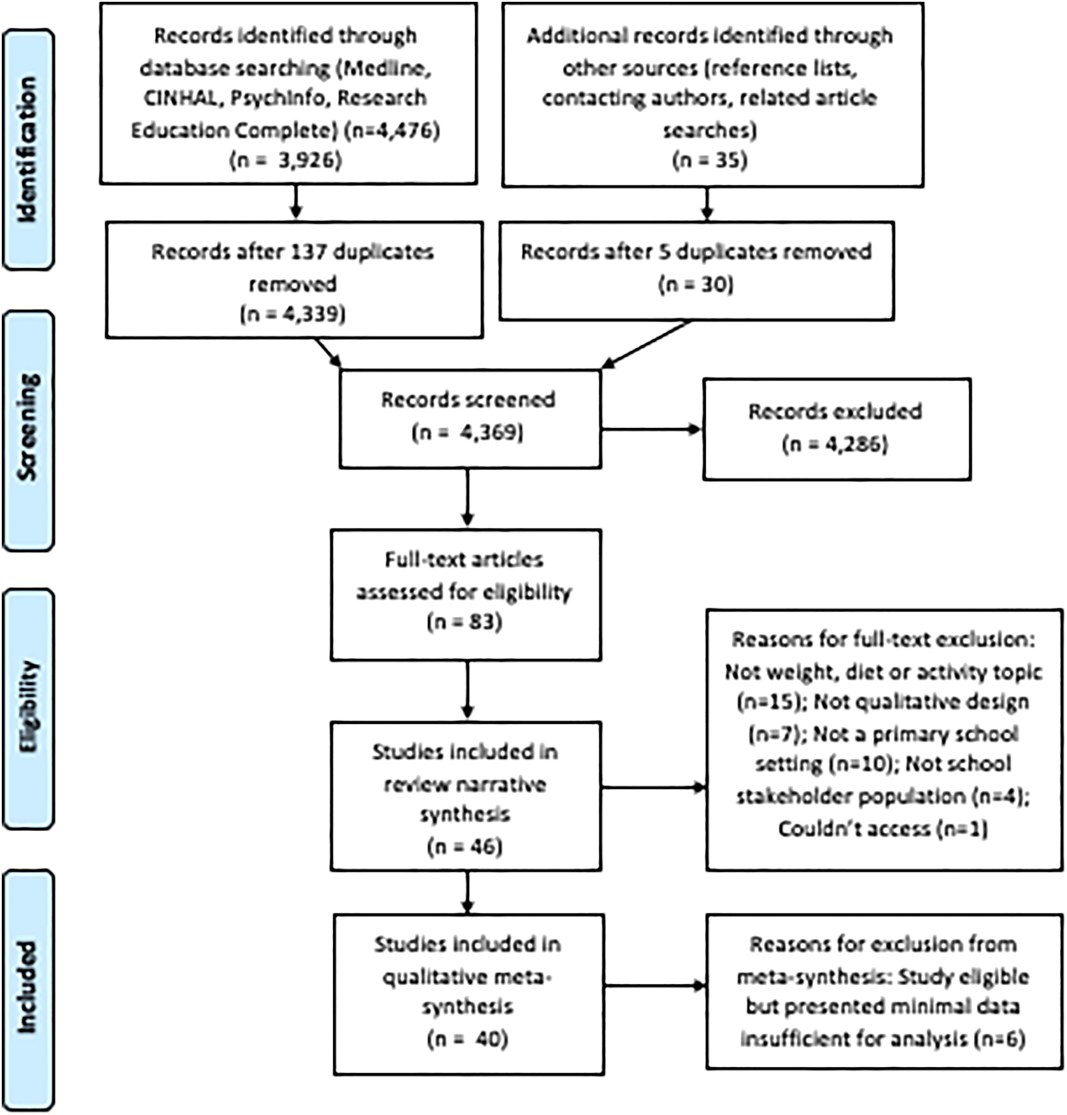
PRISMA flowchart

Following initial screening of titles and abstracts by one author (AC), three authors with prior experience of conducting systematic reviews (AC, SP, JH) independently screened full text articles deriving from database search results. Seventy‐five full texts were screened in accordance with full eligibility criteria by two of the three reviewers, and disagreements were resolved via discussion between all three reviewers where necessary. Interrater reliability scores indicated “substantial” agreement (Kappa = 0.64) between the three reviewers.^28^ See Figure 1 for PRISMA diagram displaying full screening details.

### 2.3 Data extraction and quality appraisal

Data extraction was performed by KUG and reviewed by AC by recording the following study characteristics in an Excel spreadsheet: study aim, design, participant group, setting, communication topic, data collection method, analytic approach, data source, and sample size. Included studies were assessed for quality using the Critical Appraisal Skills Programme^29^ for qualitative research as this is a well‐established and easily accessible tool allowing for procedural replication of the current study. It also includes specific items that are relevant and applicable to the current review purpose and content. It comprised of a 10‐item checklist assessing study validity, confidence in results, and likely study value. Four reviewers completed the quality appraisals for included studies, with each study being independently appraised by two of the four reviewers and any disagreements being resolved by discussion with a third reviewer where necessary. Interrater reliability was calculated on a third of included papers (*n =* 15) indicating that agreements levels were “almost perfect” (Kappa = 0.88) for overall quality appraisal.^28^

### 2.4 Analysis and synthesis

A qualitative, inductive thematic synthesis was conducted to analyze the data from included studies.^25^ Data extracted from studies included primary source data (e.g., quotes) and interpretive comments from study authors (e.g., text within results sections of papers). Data were extracted and analyzed within NVivo 10 and Microsoft Word. NVivo was used for line‐by‐line coding, memo writing, and initial organization of the thematic structure. Microsoft Word was used iteratively with NVivo to further develop the thematic analysis structure and theme and subtheme labels and definitions. Analysis involved line‐by‐line coding in which data relevant to the review research question was highlighted and annotated by one author (KUG). Descriptive and then analytic themes were identified by collating similar categories of codes within the analysis documents and holding regular discussions with another reviewer (AC) to define theme parameters and test out the evidence for each theme and subtheme. Final themes were reviewed with input from an additional reviewer (NC). Although remaining close to analyses conducted within included studies, the final analytic themes included in this review went beyond those themes generated by individual studies to identify cross‐cutting themes accounting for patterns across the wider database.

## 3 RESULTS

### 3.1 Study characteristics and quality appraisal

Forty studies (2324 participants) from the United States (*n =* 19), United Kingdom (*n =* 9), Sweden (*n =* 6), Australia (*n =* 2), Netherlands (*n =* 2), Finland (*n =* 1), and Canada (*n =* 1) were included in the meta‐synthesis. Of the 28 studies that reported gender, 26% (292/1103) of participants were male. Participant groups included parents (*n =* 9), teachers/head teachers (*n =* 4), children (*n =* 2), school nurses (*n =* 8), or a combination of two (*n =* 8) or more (*n =* 9) of these groups. Topic of communication reported in the studies included obesity and/or overweight (*n =* 15), diet and/or physical activity (*n =* 11), BMI (body mass index) screening (*n =* 7), general or weight‐related health/well‐being (*n =* 2), or a combination of two or more of these (*n =* 2). Mindful yoga (*n =* 1), eating behavior (*n =* 1) and school nurse‐child cooperation (*n =* 1) were also reported. References and information for each individual study are provided in the supporting information File S3.

With regard to quality appraisal, CASP items 1–9 are included in Table 2, showing that overall quality was high, except that the relationship between researcher and participant was not addressed (Q6) in the majority of papers (*n =* 30, 75%). Question 10 regarding the value of the research was not included in the table given answers were related to the value of the research and were nonbinary. Of these, most of the included papers were deemed valuable with regard to how well they related and contributed to existing literature, their explicit reporting of implications for research and/or practice, and novelty of approach. A minority (*n =* 4, 10%) were considered less valuable as they failed to indicate implications of their findings,^31^, ^37^ had not related their findings to existing literature,^64^ or both of these.^51^

**TABLE 2:** Appraisal of methodological quality of included studies using CASP^30^

Author	1. Was there a clear statement of the aims of the research?	2. Is a qualitative methodology appropriate?	3. Was the research design appropriate to address the aims of the research	4. Was the recruitment strategy appropriate to the aims of the research?	5. Were the data collected in a way that addressed the research issue?	6. Was the relationship between researcher and participants adequately addressed?	7. Have ethical issues been taken into account?	8. Was the data analysis sufficiently rigorous?	9. Is there a clear statement of findings?
Bartelink et al.^7^	✓	✓	✓	✓	✓	✓	?	✓	✓
Bergström et al.^31^	✓	✓	✓	✓	✓	✓	✓	✓	✓
Bergström et al.^32^	✓	✓	✓	✓	✓	×	✓	✓	✓
Booth et al.^33^	×	✓	✓	?	✓	×	✓	?	✓
Clarke et al.^34^	✓	✓	✓	✓	✓	×	✓	✓	✓
Dariotis et al.^35^	✓	✓	✓	✓	✓	×	?	✓	✓
Ganter et al.^36^	✓	✓	✓	✓	✓	✓	✓	✓	✓
Grimmet et al.^37^	✓	?	?	✓	?	×	✓	?	✓
Hall et al.^38^	✓	✓	✓	✓	✓	✓	✓	✓	✓
Hart (2003)	✓	✓	✓	✓	✓	×	✓	✓	✓
Howard‐Drake and Halliday^20^	✓	✓	✓	✓	✓	✓	✓	✓	✓
Jago et al.^39^	✓	✓	✓	✓	✓	×	✓	✓	✓
Keough (2015)^40^	✓	✓	✓	✓	✓	×	✓	✓	✓
Kipping et al.^41^	✓	✓	✓	✓	✓	×	✓	✓	✓
Kubik et al.^42^	✓	✓	✓	✓	✓	×	✓	✓	✓
Lloyd and Wyatt^43^	✓	✓	✓	✓	✓	✓	✓	✓	✓
Luesse et al.^44^	✓	✓	✓	✓	✓	×	✓	✓	✓
Mäenpää et al.^45^	✓	✓	✓	✓	✓	×	✓	✓	✓
Magnunnson et al.^46^	✓	✓	✓	✓	✓	×	✓	✓	✓
Moore et al.^47^	✓	✓	✓	✓	✓	×	✓	✓	✓
Morrison‐Sandberg et al.^48^	✓	✓	✓	✓	✓	×	✓	✓	✓
Moyer et al.^49^	✓	✓	✓	✓	✓	×	✓	?	✓
Müllersdorf et al.^21^	✓	✓	✓	✓	✓	✓	✓	✓	✓
Norman et al.^50^	✓	✓	✓	✓	✓	×	✓	✓	✓
Passmore et al.^51^	✓	✓	✓	✓	✓	×	✓	✓	✓
Powell et al.^52^	✓	✓	✓	?	?	×	✓	✓	✓
Powell et al.^30^	✓	✓	?	✓	✓	✓	✓	?	✓
Ramos and Mccullick^53^	✓	✓	✓	×	✓	✓	×	?	✓
Ruggieri and Bass^54^	✓	✓	✓	✓	✓	×	?	?	✓
Schalwijk^55^	✓	✓	✓	✓	✓	✓	✓	✓	✓
Schroeder and Smaldone^56^	✓	✓	✓	✓	✓	×	✓	✓	✓
Schwartz (2015)^57^	✓	✓	✓	✓	✓	×	✓	✓	✓
Stalter et al.^58^	✓	✓	✓	✓	✓	×	✓	✓	✓
Stalter et al.^59^	✓	✓	✓	✓	✓	×	✓	✓	✓
Steele et al.^60^	✓	✓	✓	✓	✓	×	✓	✓	✓
Thompson et al.^61^	✓	✓	✓	✓	✓	×	✓	✓	✓
Thornstensson et al.^22^	✓	✓	✓	✓	✓	×	✓	✓	✓
Turner et al.^62^	✓	✓	✓	✓	✓	×	✓	✓	✓
Tyler and Horner^63^	✓	✓	✓	✓	×	×	✓	✓	✓
Weatherson et al.^64^	✓	✓	✓	✓	✓	×	✓	✓	✓

*Note*: ✓ = yes; × = no; ? = undeterminable.

### 3.2 Meta‐synthesis results

Four themes were identified by the thematic synthesis of stakeholders' views and experiences of weight management discussions. These were (1) conversation characteristics and consequences, (2) missing components, (3) avoiding stigma, and (4) school responsibilities. Below is a full description of themes and subthemes alongside illustrative quotes from participants within the included papers, unless otherwise stated (i.e., “author interpretation”).

### 3.3 Theme 1: “Conversation characteristics and consequences”

Stakeholders described experiencing child obesity‐related conversations in school settings as possessing specific characteristics, indicating the nature of these encounters and resulting in a range of consequences for those involved in terms of their thoughts and feelings on the subject.

#### 3.3.1 Characteristics

In terms of the characteristics of these conversations, stakeholders identified encounters as being sensitive in topic, uncomfortable, yet opportunistic, *and* necessary. Stakeholders primarily highlighted experiencing high levels of sensitivity and related discomfort regarding child weight conversations. School nurses viewed discussions about children's obesity as emotionally taxing, provocative, and an intrusion on the family's private life, with a potential to insult the child and parents or evoke discomfort.^21^, ^22^, ^48^, ^59^

Previous challenging experiences of informing parents about weight‐related issues had left school nurses feeling insecure about approaching families, with both school nurses and headteachers reporting feeling intimidated and anxious about contacting parents regarding this sensitive issue.^21^, ^58^, ^59^, ^60^ For school nurses, this resulted in use of indirect methods for contact (e.g., letters) or focusing discussions around associated health issues (e.g., blood pressure).^21^, ^58^, ^60^ Parents also reported their sensitivity to such discussions,^40^ and head teachers viewed childhood obesity as a sensitive subject that felt extremely challenging to openly discuss.^20^ Furthermore, headteachers highlighted their discomfort with being unsure how to respond to judgmental encounters by children which could occur without staff being able to provide alternative viewpoints:

“*Where we do not have racist comments we do have fat comments*. *It*’*s difficult because you cannot address it*, *if she*’*s fat you cannot say she*’*s not fat*.” *(headteacher)* ^20^

Despite this sense of discomfort, stakeholders felt that parents of all children should be notified of their child's weight, though where resources were limited parents of overweight children should be proritized.^42^ Parents valued receiving notifications about their children's weight specifically because it (a) serves as direct feedback on their development, (b) enables raising any potential concerns about their child's weight/health, (c) is useful in influencing healthy behavior change, and (d) gives parents autonomy to act if necessary^37^, ^42^, ^49^: “*the majority of parents want to know [about child*’*s BMI] so they can take care of their kids*.”^42^ Children also expressed a need to be told the results so they could reflect on their own health behaviors:

“*I think it*’*s really cool coz it*’*s a chance to talk about how I feel about myself and I can find out if I need to maybe do a little more exercise or eat a little bit healthier*.” *(child)* ^37^

#### 3.3.2 Consequences

Stakeholders identified tangible outcomes of these conversations that they had observed. They reported that these encounters could impact on individuals' thoughts and feelings including children's self‐esteem and families' motivation for weight‐related behavior change.

Regarding emotional consequences, some families were receptive and accepting of being informed about children's weight status,^49^ while others were more neutral^57^ or “*really did not care*.”^56^ However, many reported a variety of negative emotions in response to child weight communication that had occurred within schools. Negative emotional consequences for parents included anger,^22^, ^34^, ^49^, ^56^, ^59^, ^60^, ^63^ upset and shame,^21^, ^37^, ^57^ and offence^22^, ^42^, ^56^, ^60^:

“*I cannot even begin to tell you the phone calls that I received … It was basically how dare I intrude …*” *(school nurse)* ^56^

Parents expressed loss of confidence due to feeling their parenting competence was questioned.^22^, ^40^ Parents' distress was also demonstrated by denial, rationalization, concern, guilt, and fear in relation to discussions about their child's weight^49^, ^57^, ^62^, ^63^:

“*You take most of the blame because you are like I*’*m the mother and I cannot believe that I did not pick healthy choices*. *Did I do enough*? *You beat yourself up and the kids notice that too*.” *(parent)* ^49^

Children with overweight or obesity also reported experiencing sadness and upset upon reading school notifications about their weight.^22^, ^37^ Emotions and attitudes also proved to be a double‐edge sword. Some parents became resistant and failed to engage with school staff about the topic or with onward referral^56^, ^62^, ^63^ or did not see the need to address weight^60^:

“*Parents do not always accept that their child has a weight issue and decline onward referral or further monitoring*” *(school nurse)* ^62^

Parents believed that, as a result of weight conversations, their children would become embarrassed, concerned about, and preoccupied with their weight negatively affecting their self‐esteem and some feared this may even contribute to the development of eating disorders.^37^, ^40^, ^49^, ^54^

“*I do not think children should be made too aware of their weight*. *Too much anorexia and bulimia around as it is*.” *(parent)* ^37^

Discussing obesity openly was identified as resulting in teasing and bullying among children^21^, ^33^, ^37^, ^42^, ^48^, ^49^, ^52^ which negatively impacted those with weight issues in terms of self‐esteem.^33^

When supportive, tailored, and person‐centered approaches were used within conversations, however, stakeholders felt more confident that families were more likely to make behavioral changes, that is, healthier eating or activity pattern.^21^ Adequate understanding of the family situation and their abilities serves as the foundation of tailored support setting highly individualized goals which in turn facilitates families' motivation and engagement,^21^, ^50^, ^63^ whereas providing advice that is too general and not specific to a particular family may not be useful.^46^

Stakeholders reported that communicating with parents and children through providing information to children or notification letters to parents resulted in them being more conscious about their habits and choices, or motivated them to continue with a healthy lifestyle.^43^, ^45^, ^49^, ^57^ Including parents in conversations was important as there was potential to motivate change in parental behavior too.^31^, ^42^

“*And it should not be just focused on the kid … Maybe it could be a wake‐up call to the parents*, *too*. ‘*Gosh*, *my child*’*s a little bit heavier*. *Maybe I am too*.” *(parent)* ^42^

Indeed, some parents believed that support should target them rather than their children.^50^

Regarding motivation, some conversations were viewed to help children and parents to make behavior changes through being listened to and articulating and achieving goals.^31^, ^50^ In their interactions with children, school nurses aimed at being “*sensitive and humble*” to increase trust, autonomy, and motivation, emphasizing “*solutions and possibilities*,” and utilizing family strengths to overcome obstacles.^22^ Another motivational strategy was to focus on preventing weight gain rather than discussing weight reduction through a positive attitude and praising without evoking guilt.^21^ A weight management intervention employed the motivational technique, “collaborative negotiation” and was delivered by trained school nurses, tailoring goals, having discussions on barriers and facilitators of goal attainment, and increasing parental mastery to promote children's health.^63^

Not only did stakeholders emphasize the potential for motivating families, but it was also reported that by staff choosing to engage about the topic of child weight in schools, this could also have a direct impact on other staff, motivating them to address the issue as well^7^, ^38^, ^50^:

“*I think because they are super excited*, *I*’*m super excited … it kind of is like a domino effect*.” *(teacher)* ^38^

### 3.4 Theme 2: “Missing components”

Despite stakeholders reporting child weight conversations being necessary (though difficult) and sometimes motivating for families, they also identified a lack of collaboration, competence, and referral options as core missing components from obesity conversations, leading to suboptimal communication between stakeholders.

#### 3.4.1 Lack of collaborative efforts

A number of studies reported a lack of collaboration between different stakeholders. While parents and teachers considered cooperation important to provide the right support to children, they both perceived their interactions insufficient. For example, parents would have preferred more follow‐up from the schools to encourage adherence to school‐based healthy weight initiatives. Teachers complained about parental failure to follow‐through with referrals to community weight management programs^60^ and not responding to education activities to promote healthy lifestyles.^31^, ^50^

“*No*, *I think we invested a lot of time and I thought I prepared a lot and it was fun with the children*, *but then got no response from the parents*.” *(teacher)* ^50^

School nurses felt that the low level of parental engagement was linked to parents' own weight or as a result of not considering excess weight of children as an issue.

“*The obesity is not such a problem then*, *they are not getting teased … a lot of them are quite cute … a bit of puppy fat…*” *(school nurse)* ^62^

Parents felt distressed and confused about the school's inadequate communication prior to weight‐related screening taking place, such as the lack of notification, lack of formal consent, and lack of confidentiality.^54^, ^57^

The absence of partnership also appeared in a wide range of other areas within the primary school context. It was uncommon that children would seek information about weight management.^60^ With regard to a mindfulness and yoga intervention, teachers felt that they would have liked more information about the progress of the children participating.^35^ School nurses expressed their concern about the lack of implementation policy for screening and information on BMI databases^58^ and the absence of follow‐up of government initiatives.^39^ Finally, despite a culture of alliance between school nurses and community health professionals in terms of a variety of different health‐related issues in children, there was a paucity of collaboration with regard to obesity and weight‐related matters.^48^

#### 3.4.2 Lack of competence

Public health awareness training for school personnel was identified as a useful resource on key health issues, including obesity.^20^ However, teachers reported insufficient training in relation to a variety of weight‐related topics,^64^ and some school nurses lacked confidence in training they had received.

“*I*’*m just hoping I*’*m doing the right thing*, *training*’*s not up to date*” *(school nurse)* ^62^

Narratives revealed a need for training on motivational approaches, child weight management, healthy eating, and physical activity.^47^, ^60^, ^62^ The lack of training in establishing relationships and talking about sensitive issues, motivating families, managing emotional and behavioral difficulties, and dealing with cultural differences were also concerns.^60^ School staff also required weight‐related health material to pass on to children and parents.^60^ Without these acquired skills and resources, teachers relied on their own experiences for example as mothers or through collegial supervision.^21^

#### 3.4.3 Lack of referral options

Although stakeholders indicated some value in notifying parents about child weight, BMI screening was also often regarded as an ineffective step without purpose: “*I mean*, *why label a kid fat without a valid plan*?”^58^ This highlighted the absence of support and onward referral options to effective treatment including community resources^59^, ^62^ and “*successful or affordable weight management programs*” (author interpretation).^58^ Furthermore, where options were available, referral was based on professional judgment resulting in inconsistent practice by staff, for example, because school nurses were working with contradictory diagnostic criteria or using visual cues about the child's weight to override centile chart results.^62^ School nurses also expressed frustration that where referrals were made, they rarely received feedback regarding outcomes.^62^

### 3.5 Theme 3: “Avoiding stigma”

Avoiding stigma was a common concern within child weight discussions, particularly around language use, confidentiality, and with regard to having a holistic approach to child health.

#### 3.5.1 Language used for weight discussions

Using appropriate language which can influence stakeholders' motivation and engagement is important given the complex and challenging nature of childhood obesity discussions.^20^ Parents reported inappropriate correspondence regarding child weight status, which evoked negative emotions,^40^, ^42^ for example,

“*Dear Ms*. *XX*, *your son has been diagnosed with obesity and we recommend that you have him see a physician and start a weight loss program immediately for his health and benefit*’. *… It was cold and totally inappropriate*.” *(parent)* ^40^

Rather, stakeholders felt communication should be respectful without generating shame in parents and children, to be able to establish effective relationships and help families feel the supportive nature of intervening.^48^ Parents expressed that children should not be diagnosed with obesity as if it was a disease or called obese and fat.^40^, ^49^ Instead, a more positive, empathetic, attentive, sensitive, and cautious discourse was advocated to avoid alienating children's or causing negative impacts on self‐esteem and self‐confidence.^21^, ^22^, ^49^, ^58^ Despite this, reports of less sensitive exchanges were recalled.

“*The nurse said little stuff like*, ‘*oh she*’*s the biggest one in the class*.’ *You*’*re not supposed to say that*.” *(parent)* ^54^

Terms such as “obese” were described by parents as “*off‐putting*” and “*derogatory*”^61^ and instead expressed that the term “at risk for overweight” was more motivating because it “*implies that something can be changed*.”^61^ On the other hand, taking a medical approach, emphasizing the potential of excess weight to lead to health problems, was considered valuable by school nurses. This included neutral terms making the condition more acceptable, generated better response, and ultimately success, such as health behavior change.^21^

#### 3.5.2 Confidentiality

Discussing weight issues at school brought unwanted attention to children's weight‐related problems in this setting.^37^ Children tell each other about their weight, which is likely to generate judgmental remarks from peers to peers.^42^

“*It*’*s bad enough being a heavyweight child let alone being embarrassed in front of the class*. ‘*Oh my god*, *she have to go [*sic*] and get a lesson from the nurse because she*’*s fat*’.” *(school nurse)* ^56^

This can be avoided if weight screening and notification are performed in a confidential manner respecting children's privacy.^42^, ^45^, ^49^, ^54^, ^56^, ^57^

“*I can get [the student] quietly in the hall and say*, ‘*Hey*, *I just want to talk to you if you get a break today*,’ *and he would say*, “*Okay*,” *and he would come back maybe after lunch or something like that*.” *(school nurse)* ^56^

Another confidentiality concern is related to attempts by schools to involve parents in child obesity interventions. Staff reported fearing that parent involvement in such activities (e.g., assisting with BMI screening) would jeopardize child confidentiality by sharing personal information with other parents.

“*There is absolutely no way I*’*d permit a parent to help with weight*. *They gossip too much…some can be hurtful even though well intended …*” *(school nurse)* ^58^

School nurses reported that some mothers who volunteered in BMI screening programs unintentionally broke confidentiality and suggested that “*rumor control*” was necessary through “*confidentiality training*” to protect confidential health information.^59^

#### 3.5.3 Holistic approach

A holistic approach to obesity was recommended by stakeholders to avoid stigma. Firstly, the use of a range of school interventions that do not specifically target obesity or overemphasize appearance would be favoured^21^, ^48^ rather than “*formal action to address obesity*” (author interpretation).^20^ These could involve activities that may be helpful in combating overweight and obesity through promoting healthy behaviors, such as active play and cooking clubs.^20^ Additionally, and similarly to the BMI notifications, school nurses expressed that obesity education should be included in the curriculum for all, to avoid labeling and “*singling out children with obesity*” (author interpretation).^56^

### 3.6 Theme 4: “School responsibilities”

Stakeholders collectively described that schools held a role in ensuring they were operating as effective communicators about the topic of child weight, diet, or physical activity and that they have their responsibility to effectively manage the logistical issues surrounding such conversations.

#### 3.6.1 Role of schools as effective communicators

This theme included key areas important to schools being effective in their communication, which incorporates clarity of communication, importance of tailoring and building rapport, and role modeling.

##### Clarity

Clear, simple, and easy to understand communication to and among stakeholders was highlighted as key in the promotion of health behaviors in children. Clarity motivated teachers and parents in their efforts and ensures accurate understanding of the information.^31^, ^42^, ^45^, ^49^, ^54^ Parents preferred short and concise communication with visual aids, such as pictures, colors, and arrows:

“*I think for people who are visual*, *[pictures are] what draws your eye first*. *Then you see your child*’*s name*, *and you see them in the overweight category*, *and then you think*, ‘*I*’*d better read this letter*.’” *(parent)* ^61^

Due to the difficulty of talking about overweight with clarity, nurses noted using tools, such as the “growth curve,” as an aid in providing an appropriate explanation to children and parents.^22^ Magnusson et al.^46^ revealed that school nurses counseling overweight children provided inadequate, unclear, and vague explanations about food and physical activity, and another study found that counseling was not straightforward enough.^45^ It has also been demonstrated that if communication is at the right level for children, it functions better.^54^At the same time, educational health materials provided to teachers can be more successfully used if it is “*informative and ready‐to‐use in class*, *with clear instructions and good structure*” (author interpretation).^50^ A common understanding and definition of foods and concepts was viewed to help facilitate behavior change by parents and ultimately in children.^44^

Finally, stakeholders reported that providing communication in families' primary language and considering their cultural background increases the quality of support as it helps avoid language and culture‐related misunderstandings.^40^, ^44^, ^46^, ^48^, ^50^, ^60^

“*We are many parents that speak Spanish*, *and many of us we can read some of it but maybe there is one word we do not understand and that changes the sentence*. *So it is important to send it in Spanish*.” *(parent)* ^44^

##### Rapport

As a way of addressing the issue of sensitivity highlighted earlier, stakeholders emphasized that a strong supportive relationship was essential. Rapport between children and school nurses in particular was deemed to help children feel comfortable and initiate conversations about weight in school settings.^45^, ^60^ From the children's perspective “*When the school nurse is familiar there is no need to fear*, *which makes it easy to approach her*.”^45^

However, discussions between school nurses and children have also been described as lacking reciprocity due to nurses' not always demonstrating listening or patience with individuals.^45^Listening to children's verbal and nonverbal cues was elsewhere reported to help customize support,^64^ but the lack of nurse contact with children outside of urgent health needs was identified as a barrier to building meaningful rapport.^60^

##### Role modeling

Role modeling was considered an important factor to influence motivation and engagement in health behavior change. Stakeholders believed that if school staff struggled with their own weight, this could negatively impact on their confidence to support others and might even reduce the receptiveness and acceptance of the support offered.^34^, ^39^, ^60^

“*I personally get the feeling that the parents are thinking*, ‘*how can you be talking about this*, *you are fat yourself*.’ “ *(school nurse)* ^60^

“*Well the teachers always tell us off for having chocolate or sweets or anything but it*’*s really kind of like*, *they are kind of not doing that themselves because at break time they always get to have cakes*” *(child)* ^39^

The importance of the idea that you “practice what you preach” was also evident in terms of physical activity education (PE). A PE teacher was only considered credible by students if they set a good example and demonstrated how healthy behaviors, such as eating and physical activity, can be incorporated into one's life. This can convince students that they could be successful in their efforts.

“*When they are teaching about how to take care of your body*, *you do not go see them do the exact opposite*.” *(child)* ^53^

#### 3.6.2 Role in managing logistics of child weight discussions

Stakeholders viewed the schools' role to be managing the logistics of child weight conversations both preceding and following such conversations. Within this theme, four areas of focus were highlighted: initiation of communication, importance of leadership and teamwork, use of appropriate communication platforms, and education and resource.

##### Initiation of communication

While stakeholders highlighted the necessity of child weight conversations, the question remained regarding whose job is it to initiate communication with parents regarding their child's weight. School nurses and teachers believed that it is their responsibility to make the first step and contact parents if their child was overweight or was experiencing a weight‐related problem (e.g., poor diet, low engagement with physical activity) and are also concerned that they may be stepping over the line.^21^, ^38^

While some parents were neutral about school staff initiating these conversations,^54^ many insisted that this information should not be coming from schools unless the child's weight interferes with school activities.^33^, ^37^, ^40^, ^49^, ^54^ Rather, they preferred their general practitioner to raise this issue,^33^, ^37^, ^49^, ^54^ with communication from school nurses focusing on medical needs only.

“*I [parent] understand that you are there for an emergency or to give out medications*, *but I do not want you to speak to my child again about nutrition*.” *(school nurse)* ^56^

However, conversations related to child weight in school settings were also found helpful in drawing the stakeholders' attention to a critical problem^64^ and it enabled support from overweight school peers.^55^ Moreover, some parents appreciated the school's support in communicating weight problems as this seemed to strengthen the information with authority resulting in greater responsiveness of children.^43^ Similarly, different stakeholders found that bringing an “outsider” into weight conversations, such as dietary experts, added capacity to their expertise and increased credibility.^34^, ^39^ This was valuable in increasing child engagement and role modeling.^39^

##### Leadership and teamwork

Collaboration among stakeholders and having strong leadership were recognized as important aspects of changing health behaviors. Alliance with other parents with similar experiences and health care providers (nurses, dieticians, personal trainers, and school lunch staff) was seen to provide additional support to parents,^57^ and stakeholders felt that if parents and teachers worked together, this reinforces the message about the importance of a healthy lifestyle to children.^22^, ^38^, ^50^, ^56^, ^58^

Working as a team with parents could therefore promote their children's health through acknowledging the parents' expertise and improving parenting.^63^ Effective teamwork among school staff was therefore believed to ensure the smooth operation of weight‐related programs as colleagues could help nurses reinforce health messages, give availability to children, and solve conflicts with parents.^56^, ^58^

“*And [the teachers] are very receptive … That helps a lot*. *I do not have any of the teachers saying* ‘*Oh*, *you cannot take them out of class*’” *(school nurse)* ^56^

Although not resistant, not all teachers were interested in getting involved themselves for example, via related training opportunities,^64^ and school staff highlighted that teamwork only functions well with effective leadership as it encourages participation, brings people together, helps facilitate interest, and aids with organization and decision making^39^, ^59^, ^64^: “*It felt like it was allowed to slide a bit after a while to be honest*.”^50^ Furthermore, leadership was reported to only be effective if teachers' autonomy is also ensured within this.

“*Telling someone you have to do something*, *changes it*. *I think if you do not have to do it*, *sometimes you are more willing to do it … the* ‘*Big Brother method*’ *does not work well*.” *(teacher)* ^64^

##### Communication platform

Parents considered face‐to‐face communication essential, but support over the phone or in an email was also acceptable^31^, ^40^ except for counseling sessions.^50^ Calling parents on the phone was found effective in delivering information about school activities and cover a variety of issues. Newsletters and sending handouts were considered a simple way to communicate by both teachers and parents^36^, ^41^, ^48^ and perhaps a form of communication that reduces barriers between teachers and parents.^38^

In terms of receiving sensitive weight‐related information about children, parents welcomed individualized letters, although parents stressed these should never be sent home with their child (unless the envelope is sealed), but ought to be posted directly to them to ensure confidentiality and flexibility to share with other family members.^40^, ^42^, ^49^, ^54^, ^57^

“*They come home with these notes saying I*’*m fat more or less*, *and they know how to read; they are not oblivious*, *and that*’*s embarrassing…*. *for me*, *it*’*s not appropriate to send a letter home saying your son is overweight*.” *(parent)* ^40^

Report cards were also not supported in case children could access the information without parental supervision.^42^ For general communication from schools on weight‐related topics, parents suggested websites and social media,^44^ information booklets^21^ as long as they were not too basic,^50^ the education curriculum,^39^ and teacher–parent conferences,^42^ as alternatives, but they were divided about receiving text messages.^44^

##### Education and information

Stakeholders discussed the different types of and delivery approaches to information required to educate children and parents about healthy lifestyles. Educational programs on promotion of diet, health, and physical activity were reported to guide nurses in dealing with childhood obesity.^21^, ^56^ However, due to time constraints, school nurses found it difficult to provide additional weight‐related education to students.^60^ A solution for this was suggested to be the integration of additional diet and physical activity related lessons into the curriculum.^39^, ^43^ As long as the materials for these lessons are clear, easy to follow, and provides flexibility to teachers, staff felt it would not be taxing or disruptive to the curriculum.^43^, ^51^

Generally, parents wanted more health behavior‐related information and resources,^57^ suggestions regarding diet and physical activity and information on how to calculate BMI.^42^, ^61^ They considered healthy cooking techniques, inexpensive recipes, and information on healthy portion size valuable.^44^ However, parents' needs regarding type of information differed depending on socioeconomic status. For example, parents from lower socioeconomic groups were in need of more basic information and advice on nutrition and practical cooking skills.^65^ Those with higher socioeconomic status were interested in more nuanced dietary information.

“*Information requested by this subgroup [high SES parents] was often very advanced*, *such as age and gender specific dietary reference values and information regarding maximizing the nutrient content of foods through alternative cooking methods and food combinations*.” *(author interpretation of parent data)* ^65^

Strategies to engage children and improve their health‐related learning within schools included practical activities, individual and interactive group approaches, reinforcement, using experiments, visuals and videos, opportunity to ask questions, and diverse materials.^38^, ^45^ Ramos et al.^53^ found that children were viewed to respond better if PE lessons included demonstrations, examples, and feedback during activities and if the teacher was articulate and participated in activities.

## 4 DISCUSSION

Answering our central research question, primary school stakeholders recognized that schools were well‐positioned to provide positive influential messages about child weight, and staff reported that discussions on this topic do occur in primary school settings. However, though viewed as necessary, this review reveals that the occurrence of these important conversations is limited due to their sensitivity and complexity. Furthermore, when conversations do occur, they are limited in terms of their effectiveness on intended outcomes, such as weight‐related behavior change. Answering our first subquestion, barriers to successful conversation included concerns over consequences, avoiding stigma, and lack of training by teaching staff. Facilitators to effective conversations included providing a holistic approach tailored to the individual child or family, by motivated staff. Regarding the second subquestion, schools were viewed as having specific roles in addressing child weight. Firstly, schools should be effective communicators, which requires clear messaging, an ability to build rapport, as well as role modeling by staff. Schools were also viewed as responsible for logistics surrounding conversations, which requires effective leadership and teamwork from school staff, effectively using an appropriate communication platform, and using and providing useful and effective resources. Some clarity is needed regarding roles in initiating conversations and taking these forward, given some differences in views between school staff and parents.

Previous findings suggest that the quality of conversations held by primary school stakeholders including children, parents, and educators is likely to be important in determining a child's ability to successfully manage their weight or make weight‐related behavioral changes.^66^ In line with our findings, a systematic review of 13 qualitative studies identified knowledge and competence as key facilitators to healthcare professionals discussing child weight with parents.^23^ In particular, our results suggest that additional training may be required for teachers and trainee teaching staff to develop these, which supports previous suggestions that teachers lack training in public health that could be relevant to their roles.^67^ Lack of clarity with regard to the role of teaching staff in public health and the need to improve their confidence and competence in dealing with issues relevant to the primary school setting is also supported by previous findings.^68^ A systematic review of 20 studies of teacher training in health and well‐being promotion identified that such training can increase teachers' factual knowledge of health issues, as well as increase confidence in teaching and helping children with specific health challenges.^69^ Furthermore, such training resulted in an increase in teachers' positive beliefs about their role in promoting child health. However, Shepherd et al.^69^ concluded that some teachers still lacked confidence and knowledge in addressing more sensitive health topics, suggesting further training is necessary for more challenging issues.

Given school nurses have more defined roles in managing child health issues, they have more specialist training and resources for weight management communication, further training may not be required. For example, in the United Kingdom, school nurses are responsible for measuring the weight and height of children in school and calculate and feedback their BMI to parents.^70^ Given the expectation to communicate with parents regarding weight, guidance is available for nurses which provides information on expectations around parental reactions, which has been identified as a barrier to communication,^23^ and a clear conversation framework to guide discussions.^71^ While further training may be unnecessary for nurses, other considerations for improving child–nurse communication not identified in this present study include the influence of the physical environment, for example, being seated at a desk in the health office,^72^ and organizational considerations, such as nurses being based within the schools, rather than being an occasional visitor.^73^ Indeed, Ofsted,^12^ the governing body responsible for school inspections in the United Kingdom highlighted in their recent report that access to school nurses was limited and sporadic, a clear barrier to communication between schools and stakeholders with more specialist skills.

It is notable that this review contained limited studies with children, and no studies identified from middle‐ and low‐income countries. This suggests that further research in these areas is needed to more fully identify existing practices and barriers to child weight interactions in primary school settings across the globe. Our findings are therefore limited to suggesting that training provision is needed for school staff from high‐income countries in initiating and holding effective weight‐related conversations with children, and research is needed to inform best practice on designing, implementing, and evaluating such training. As the broader literature also identifies mixed evidence for school‐based obesity interventions with often only small effects in weight reductions in high‐income countries,^74^ further research is also needed to determine those interventions that can achieve large and more generalisable effects. These previous types of school‐based interventions also tend to focus on increasing engagement with school‐day physical activity and/or healthy eating, rather than improving stakeholder communication. As this systematic review has identified distance between parent and staff perspectives, and lack of staff confidence and skill in raising the topic with parents, it would be valuable to design support resources for staff that focus on raising and discussing the topic of child weight with parents in a manner that is appropriate to the school context (e.g., issues of confidentiality) and to staff roles (e.g., within their occupational remit). Interventions are also more likely to be successful if they include parents in the intervention development and implementation^11^ and in the intervention itself^17^, ^75^; thus, work should move further towards designing and testing interventions that actively involve parents as key stakeholders. Previous evidence supports the current findings that the level of discomfort experienced by those involved in discussing weight management can lead to avoidance of the conversation, jeopardizing opportunity for intervention. For example, health professionals may distract, minimize, or shut down conversations they feel have the potential to insult or upset their patients.^76^, ^77^ This is therefore a key area for future interventions to address.

## 5 CONCLUSIONS

Primary school stakeholders recognized that they are well‐positioned to provide positive influential messages about child weight and reported that discussions on this topic do occur in primary school settings. However, conversations about child weight were identified as difficult and complex, and stakeholders sometimes lack the necessary skills to deliver the necessary nonjudgmental, consistent, and tailored support. This highlights a clear need for educator support in initiating these conversations and enhancing their quality to make weight‐related behavior change more likely to occur and be maintained. Training for school staff in particular may be required, which should aim to include: greater clarity of roles, language use, confidentiality, clear messaging, rapport, holistic approach, initiating sensitive conversations, clear route for follow‐on support, and bidirectional feedback on outcomes of the actions taken by stakeholders.

## CONFLICT OF INTEREST

This work was funded by the Academy of Medical Sciences which was payable to the University of Liverpool and from which NC received salary payments.

## Supporting information


**Data S1.** Supporting Information

**Table S1.** Template free‐text search terms and Boolean operators (subsequently adapted to specific database filters see below)

**Data S2.** Supporting Information
