# Supplementary material for: Exploring primary school years interactions around child weight: A qualitative meta‐synthesis of school staff, parent, and child views and experiences
Source: Obes Rev. 2022 Apr 10;23(8):e13451. doi: 10.1111/obr.13451 (PMC9539573; doi:10.1111/obr.13451)
Supplement: Supplementary file 1 — Data S1. Supporting Information [file OBR-23-e13451-s002.pdf]

Supporting Information – Supplementary Files

Title: Exploring primary school years interactions around child weight: A qualitative meta-synthesis of school staff, parent and child views and experiences.

Authors: Anna Chisholm[a], Nia Coupe[a], Katalin Ujhelyi Gomez[a], Jo Hart[b], & Sarah Peters[c]

Affiliations: [a]Department of Psychology, University of Liverpool, Liverpool, UK; [b]Division of Medical; Education, University of Manchester, Manchester UK; [c]Division of Psychology and Mental Health, University of Manchester, Manchester UK

Supplementary File 1: PRISMA & ENREQ checklists

PRISMA checklist

Section/topic	#	Checklist item	Reported on page #
**TITLE**			
Title	1	Identify the report as a systematic review, meta-analysis, or both.	Title page (meta-synthesis)
**ABSTRACT**			
Structured summary	2	Provide a structured summary including, as applicable: background; objectives; data sources; study eligibility criteria, participants, and interventions; study appraisal and synthesis methods; results; limitations; conclusions and implications of key findings; systematic review registration number.	1
**INTRODUCTION**			
Rationale	3	Describe the rationale for the review in the context of what is already known.	2-4
Objectives	4	Provide an explicit statement of questions being addressed with reference to participants, interventions, comparisons, outcomes, and study design (PICOS).	4
**METHODS**			
Protocol and registration	5	Indicate if a review protocol exists, if and where it can be accessed (e.g., Web address), and, if available, provide registration information including registration number.	1, 5
Eligibility criteria	6	Specify study characteristics (e.g., PICOS, length of follow-up) and report characteristics (e.g., years considered, language, publication status) used as criteria for eligibility, giving rationale.	5, Table 1
Information sources	7	Describe all information sources (e.g., databases with dates of coverage, contact with study authors to identify additional studies) in the search and date last searched.	5
Search	8	Present full electronic search strategy for at least one database, including any limits used, such that it could be repeated.	Supplementary file 2
Study selection	9	State the process for selecting studies (i.e., screening, eligibility, included in systematic review, and, if applicable, included in the meta-analysis).	6
Data collection process	10	Describe method of data extraction from reports (e.g., piloted forms, independently, in duplicate) and any processes for obtaining and confirming data from investigators.	6
Data items	11	List and define all variables for which data were sought (e.g., PICOS, funding sources) and any assumptions and simplifications made.	n/a
Risk of bias in individual studies	12	Describe methods used for assessing risk of bias of individual studies (including specification of whether this was done at the study or outcome level), and how this information is to be used in any data synthesis.	6
Summary measures	13	State the principal summary measures (e.g., risk ratio, difference in means).	n/a

Synthesis of results	14	Describe the methods of handling data and combining results of studies, if done, including measures of consistency (e.g., I^2) for each meta-analysis.	n/a

Section/topic	#	Checklist item	Reported on page #
Risk of bias across studies	15	Specify any assessment of risk of bias that may affect the cumulative evidence (e.g., publication bias, selective reporting within studies).	7
Additional analyses	16	Describe methods of additional analyses (e.g., sensitivity or subgroup analyses, meta-regression), if done, indicating which were pre-specified.	n/a
**RESULTS**			
Study selection	17	Give numbers of studies screened, assessed for eligibility, and included in the review, with reasons for exclusions at each stage, ideally with a flow diagram.	Figure 1.
Study characteristics	18	For each study, present characteristics for which data were extracted (e.g., study size, PICOS, follow-up period) and provide the citations.	Supplementary file 3
Risk of bias within studies	19	Present data on risk of bias of each study and, if available, any outcome level assessment (see item 12).	7, Table 2.
Results of individual studies	20	For all outcomes considered (benefits or harms), present, for each study: (a) simple summary data for each intervention group (b) effect estimates and confidence intervals, ideally with a forest plot.	n/a
Synthesis of results	21	Present results of each meta-analysis done, including confidence intervals and measures of consistency.	n/a
Risk of bias across studies	22	Present results of any assessment of risk of bias across studies (see Item 15).	7, Table 2.
Additional analysis	23	Give results of additional analyses, if done (e.g., sensitivity or subgroup analyses, meta-regression [see Item 16]).	n/a
**DISCUSSION**			
Summary of evidence	24	Summarize the main findings including the strength of evidence for each main outcome; consider their relevance to key groups (e.g., healthcare providers, users, and policy makers).	21-22
Limitations	25	Discuss limitations at study and outcome level (e.g., risk of bias), and at review-level (e.g., incomplete retrieval of identified research, reporting bias).	23-24
Conclusions	26	Provide a general interpretation of the results in the context of other evidence, and implications for future research.	23-24
**FUNDING**			
Funding	27	Describe sources of funding for the systematic review and other support (e.g., supply of data); role of funders for the systematic review.	Title page

From: Moher D, Liberati A, Tetzlaff J, Altman DG, The PRISMA Group (2009). Preferred Reporting Items for Systematic Reviews and Meta-Analyses: The PRISMA Statement. PLoS Med 6(7): e1000097. doi:10.1371/journal.pmed1000097

ENTREQ checklist

Item	Guide and description	Reported on page #
Aim	State the research question the synthesis addresses	4
Synthesis methodology	Identify the synthesis methodology or theoretical framework which underpins the synthesis, and describe the rationale for choice of methodology (e.g. meta- ethnography, thematic synthesis, critical interpretive synthesis, grounded theory synthesis, realist synthesis, meta-aggregation, meta-study, framework synthesis).	5
Approach to searching	Indicate whether the search was pre-planned (comprehensive search strategies to seek all available studies) or iterative (to seek all available concepts until theoretical saturation is achieved).	5
Inclusion criteria	Specify the inclusion/exclusion criteria (e.g. in terms of population, language, year limits, type of publication, study type).	Page 5, Table 1
Data sources	Describe the information sources used (e.g. electronic databases (MEDLINE, EMBASE, CINAHL, psychINFO, Econlit), grey literature databases (digital thesis, policy reports), relevant organisational websites, experts, information specialists, generic web searches (Google Scholar), hand searching, reference lists) and when the searches were conducted; provide the rationale for using the data sources.	5
Electronic search strategy	Describe the literature search (e.g. provide electronic search strategies with population terms, clinical or health topic terms, experiential or social phenomena related terms, filters for qualitative research and search limits).	5, Supplementary file 2
Study screening methods	Describe the process of study screening and sifting (e.g. title, abstract and full text review, number of independent reviewers who screened studies)	6
Study characteristics	Present the characteristics of the included studies (e.g. year of publication, country, population, number of participants, data collection, methodology, analysis, research questions).	7, and supplementary file 3
Study selection results	Identify the number of studies screened and provide reasons for study exclusion (e.g. for comprehensive searching, provide numbers of studies screened and reasons for exclusion indicated in a figure/flowchart; for iterative searching describe reasons for study exclusion and inclusion based on modifications to the research question and/or contribution to theory development).	Figure 1
Rationale for appraisal	Describe the rationale and approach used to appraise the included studies or selected findings (e.g. assessment of conduct (validity and robustness), assessment of reporting (transparency), assessment of content and utility of the findings).	6

Appraisal items	State the tools, frameworks and criteria used to appraise the studies or selected findings (e.g. Existing tools: CASP, QARI, COREQ, Mays and Pope [25]; reviewer developed tools; describe the domains assessed: research team, study design, data analysis and interpretations, reporting).	6
Appraisal results	Present results of the quality assessment and indicate which articles, if any, were weighted/excluded based on the assessment and give the rationale.	7-8, Table 2
Data extraction	Indicate which sections of the primary studies were analysed and how were the data extracted from the primary studies? (e.g. all text under the headings "results /conclusions" were extracted electronically and entered into a computer software).	7
Software	State the computer software used, if any.	7
Number of reviewers	Identify who was involved in coding and analysis.	7
Coding	Describe the process for coding of data (e.g. line by line coding to search for concepts).	7
Study comparison	Describe how were comparisons made within and across studies (e.g. subsequent studies were coded into pre-existing concepts, and new concepts were created when deemed necessary).	7
Derivation of themes	Explain whether the process of deriving the themes or constructs was inductive or deductive.	7
Quotations	Provide quotations from the primary studies to illustrate themes/constructs, and identify whether the quotations were participant quotations or the author's interpretation.	8-21
Synthesis output	Present rich, compelling and useful results that go beyond a summary of the primary studies (e.g. new interpretation, models of evidence, conceptual models, analytical framework, development of a new theory or construct).	Table 3, 8-21

Supplementary File 2: Search Strategy

Template free-text search terms and Boolean operators (subsequently adapted to specific database filters see below)

**Boolean operators**		**AND**	**AND**	**AND**	**AND**
**Search terms**	stakeholder OR parent* OR mother OR father OR guardian OR teacher OR head teacher OR headteacher OR principal OR teaching assistant OR student OR pupil OR child* OR school nurs* OR governor OR famil* OR adolescent OR pediatric* OR paediatric* OR dietic* OR educat* OR nutritionist	communicat* OR conversation* OR discuss* OR talk* OR interaction* OR dialogue OR chat	weight OR obes* OR overweight OR growth OR diet OR physical activ* OR exercise* OR body mass	school* OR primary school OR infant school OR elementary school	Interview* OR focus group* OR qualitative

Ovid MEDLINE (hits = 258, searched 25-04-19)

1. (stakeholder or parent* or mother or father or guardian or teacher or head teacher or principal or teaching assistant or student or pupil or child* or school* or nurse or governor or famil* or adolescent or pediatric* or paediatric* or dietic* or educat* or nutritionist).mp. [mp=title, abstract, original title, name of substance word, subject heading word, floating sub-heading word, keyword heading word, organism supplementary concept word, protocol supplementary concept word, rare disease supplementary concept word, unique identifier, synonyms]
2. limit 1 to (english language and full text and humans)
3. (communicat* or conversation* or discuss* or talk* or interaction* or dialogue or chat).mp. [mp=title, abstract, original title, name of substance word, subject heading word, floating sub-heading word, keyword heading word, organism supplementary concept word, protocol supplementary concept word, rare disease supplementary concept word, unique identifier, synonyms]
4. limit 3 to (english language and full text and humans)
5. (weight or obes* or overweight or growth or diet or physical activ* or exercise* or body mass).mp. [mp=title, abstract, original title, name of substance word, subject heading word, floating sub-heading word, keyword heading word, organism supplementary concept word, protocol supplementary concept word, rare disease supplementary concept word, unique identifier, synonyms]
6. limit 5 to (english language and full text and humans)
7. (school* or primary school or infant school or elementary school).mp. [mp=title, abstract, original title, name of substance word, subject heading word, floating sub-heading word, keyword heading word, organism supplementary concept word, protocol supplementary concept word, rare disease supplementary concept word, unique identifier, synonyms]
8. limit 7 to (english language and full text and humans)
9. (Interview* or focus group* or qualitative).mp. [mp=title, abstract, original title, name of substance word, subject heading word, floating sub-heading word, keyword heading word, organism supplementary concept word, protocol supplementary concept word, rare disease supplementary concept word, unique identifier, synonyms]
10. limit 9 to (english language and full text and humans)
11. 2 and 4 and 6 and 8 and 10

EBSCOhost PsycINFO (hits = 1,650, searched 19-06-19)

TX (stakeholder OR parent* OR mother OR father OR guardian OR teacher OR head teacher OR headteacher OR principal OR teaching assistant OR student OR pupil OR child* OR school nurs* OR governor OR famil* OR adolescent OR pediatric* OR paediatric* OR dietic* OR educat* OR nutritionist) AND TX (communicat* OR conversation* OR discuss* OR talk* OR interaction* OR dialogue OR chat) AND TX (weight OR obes* OR overweight OR growth OR diet OR physical activ* OR exercise* OR body mass) AND TX (school* OR primary school OR infant school OR elementary school) AND TX (Interview* OR focus group* OR qualitative). Search mode: Find all my search terms. Limits: full-text online; English language.

EBSCOhost CINHAL Plus (hits = 363, searched 24-06-19)

(stakeholder OR parent* OR mother OR father OR guardian OR teacher OR head teacher OR headteacher OR principal OR teaching assistant OR student OR pupil OR child* OR school nurs* OR governor OR famil* OR adolescent OR pediatric* OR paediatric* OR dietic* OR educat* OR nutritionist) AND (communicat* OR conversation* OR discuss* OR talk* OR interaction* OR dialogue OR chat) AND (weight OR obes* OR overweight OR growth OR diet OR physical activ* OR exercise* OR body mass) AND (school* OR primary school OR infant school OR elementary school) AND (Interview* OR focus group* OR qualitative): Search mode Boolean/Phrase. Limits: English language, academic journals.

EBSCOhost Education Research Complete (hits = 1,655, searched 15-07-19)

(stakeholder OR parent* OR mother OR father OR guardian OR teacher OR head teacher OR headteacher OR principal OR teaching assistant OR student OR pupil OR child* OR school nurs* OR governor OR famil* OR adolescent OR pediatric* OR paediatric* OR dietic* OR educat* OR nutritionist) AND (communicat* OR conversation* OR discuss* OR talk* OR interaction* OR dialogue OR chat) AND (weight OR obes* OR overweight OR growth OR diet OR physical activ* OR exercise* OR body mass) AND (school* OR primary school OR infant school OR elementary school) AND (Interview* OR focus group* OR qualitative): Search mode: Boolean/Phrase, Limits: English Language.

Supplementary File 3: Included study references and table of characteristics

References for studies included in review

Bartelink, N., van Assema, P., Jansen, M., Savelberg, H., Moore, G., Hawkins, J., & Kremers, S. (2019). Process evaluation of the healthy primary School of the Future: the key learning points. BMC public health, 19(1), 698.

Bergström, H., Haggård, U., Norman, Å., Sundblom, E., Elinder, L. S., & Nyberg, G. (2015). Factors influencing the implementation of a school-based parental support programme to promote health-related behaviours—interviews with teachers and parents. BMC public health, 15(1), 541.

Bergström, H., Sundblom, E., Elinder, L. S., Norman, Å., & Nyberg, G. (2020). Managing Implementation of a Parental Support Programme for Obesity Prevention in the School Context: The Importance of Creating Commitment in an Overburdened Work Situation, a Qualitative Study. The Journal of Primary Prevention, 1-19.

Booth, M. L., King, L. A., Pagnini, D. L., Wilkenfeld, R. L., & Booth, S. L. (2009). Parents of school students on childhood overweight: the Weight of Opinion Study. Journal of paediatrics and child health, 45(4), 194-198.

Clarke, J., Pallan, M., Lancashire, E., & Adab, P. (2017). Obesity prevention in English primary schools: headteacher perspectives. Health promotion international, 32(3), 490-499.

Dariotis, J. K., Mirabal-Beltran, R., Cluxton-Keller, F., Feagans Gould, L., Greenberg, M. T., & Mendelson, T. (2017). A qualitative exploration of implementation factors in a school-based mindfulness and yoga program: Lessons learned from students and teachers. Psychology in the Schools, 54(1), 53-69.

Ganter, C., Aftosmes-Tobio, A., Chuang, E., Blaine, R. E., Land, T., & Davison, K. K. (2016). Community stakeholders' perceptions of major factors influencing childhood obesity, the feasibility of programs addressing childhood obesity, and persisting gaps. Journal of community health, 41(2), 305-314.

Grimmett, C., Croker, H., Carnell, S., & Wardle, J. (2008). Telling parents their child's weight status: psychological impact of a weight-screening program. Pediatrics, 122(3), e682-e688.

Hall, E., Chai, W., & Albrecht, J. A. (2016). A qualitative phenomenological exploration of teachers' experience with nutrition education. American Journal of Health Education, 47(3), 136-148.

Hart, K., Herriot, A., Bishop, J., & Truby, H. (2003). Promoting healthy diet and exercise patterns amongst primary school children: a qualitative investigation of parental perspectives. Journal of Human Nutrition and Dietetics, 16(2), 89-96.

Howard-Drake, E., & Halliday, V. (2016). Exploring primary school headteachers' perspectives on the barriers and facilitators of preventing childhood obesity. Journal of Public Health, 38(1), 44-52.

Jago, R., Rawlins, E., Kipping, R., Wells, S., Chittleborough, C., Peters, T., . . . Campbell, R. (2015). Lessons learned from the AFLY5 RCT process evaluation: implications for the design of physical activity and nutrition interventions in schools. BMC public health, 15(1), 1-10.

Keough, L. (2015). Caregivers of Underserved Minority Populations: Views and Opinions of the Role of Schools in BMI Screening, Education and Communication. Journal of Health Disparities Research & Practice, 8(3).

Kipping, R., Jago, R., & Lawlor, D. (2012). Developing parent involvement in a school-based child obesity prevention intervention: a qualitative study and process evaluation. Journal of Public Health, 34(2), 236-244.

Kubik, M. Y., Story, M., & Rieland, G. (2007). Developing school-based BMI screening and parent notification programs: findings from focus groups with parents of elementary school students. Health Education & Behavior, 34(4), 622-633.

Lloyd, J. J., & Wyatt, K. M. (2014). Qualitative findings from an exploratory trial of the Healthy Lifestyles Programme (HeLP) and their implications for the process evaluation in the definitive trial. BMC public health, 14(1), 578.

Luesse, H. B., Paul, R., Gray, H. L., Koch, P., Contento, I., & Marsick, V. (2018). Challenges and Facilitators to Promoting a Healthy Food Environment and Communicating Effectively with Parents to Improve Food Behaviors of School Children. Maternal and Child Health Journal, 22(7), 958-967. doi:10.1007/s10995-018-2472-7

Mäenpää, T., Paavilainen, E., & Åstedt-Kurki, P. (2007). Cooperation with school nurses described by Finnish sixth graders. International Journal of Nursing Practice, 13(5), 304-309. doi:10.1111/j.1440-172X.2007.00642.x

Magnusson, M. B., Kjellgren, K. I., & Winkvist, A. (2012). Enabling overweight children to improve their food and exercise habits – school nurses' counselling in multilingual settings. Journal of clinical nursing, 21(17-18), 2452-2460. doi:10.1111/j.1365-2702.2012.04113.x

Moore, S. N., Tapper, K., & Murphy, S. (2010). Feeding strategies used by primary school meal staff and their impact on children's eating. Journal of Human Nutrition and Dietetics, 23(1), 78-84. doi:10.1111/j.1365-277X.2009.01009.x

Morrison-Sandberg, L. F., Kubik, M. Y., & Johnson, K. E. (2011). Obesity Prevention Practices of Elementary School Nurses in Minnesota:Findings From Interviews With Licensed School Nurses. The Journal of School Nursing, 27(1), 13-21. doi:10.1177/1059840510386380

Moyer, L. J., Carbone, E. T., Anliker, J. A., & Goff, S. L. (2014). The Massachusetts BMI letter: A qualitative study of responses from parents of obese children. Patient education and counseling, 94(2), 210-217. doi:https://doi.org/10.1016/j.pec.2013.10.016

Müllersdorf, M., Zuccato, L. M., Nimborg, J., & Eriksson, H. (2010). Maintaining professional confidence – monitoring work with obese schoolchildren with support of an action plan. Scandinavian Journal of Caring Sciences, 24(1), 131-138. doi:10.1111/j.1471-6712.2009.00696.x

Norman, Å., Nyberg, G., Elinder, L. S., & Berlin, A. (2016). One size does not fit all–qualitative process evaluation of the Healthy School Start parental support programme to prevent overweight and obesity among children in disadvantaged areas in Sweden. BMC public health, 16(1), 37. doi:10.1186/s12889-016-2701-1

Passmore, E., Donato-Hunt, C., Maher, L., Havrlant, R., Hennessey, K., Milat, A., & Farrell, L. (2017). Evaluation of a pilot school-based physical activity challenge for primary students. Health Promotion Journal of Australia, 28(2), 103-109.

Powell, S. B., Engelke, M. K., & Neil, J. A. (2018b). Seizing the moment: Experiences of school nurses caring for students with overweight and obesity. The Journal of School Nursing, 34(5), 380-389.

Powell, S. B., Engelke, M. K., & Swanson, M. S. (2018a). Quality of Life in School-Age Children with Obesity.

Ramos, N. C., & Mccullick, B. A. (2015). Elementary students' construct of physical education teacher credibility. Journal of teaching in physical education, 34(4), 560-575.

Ruggieri, D. G., & Bass, S. B. (2016). African-American Parents' knowledge and perceptions about BMI measurements, school-based BMI screening programs, and BMI report cards: results from a qualitative investigation and implications for school-to-parent communication. Journal of racial and ethnic health disparities, 3(2), 320-330.

Schalkwijk, A., Bot, S., De Vries, L., Westerman, M., Nijpels, G., & Elders, P. (2015). Perspectives of obese children and their parents on lifestyle behavior change: a qualitative study. International Journal of Behavioral Nutrition and Physical Activity, 12(1), 1-8.

Schroeder, K., & Smaldone, A. (2017). What barriers and facilitators do school nurses experience when implementing an obesity intervention? The Journal of School Nursing, 33(6), 456-466.

Schwartz, M. (2015). Parental perceptions of body mass index notification: a qualitative study. Journal of School Health, 85(10), 714-721.

Stalter, A. M., Chaudry, R. V., & Polivka, B. J. (2010). Facilitating factors and barriers to BMI screening in schools. The Journal of School Nursing, 26(4), 320-330.

Stalter, A. M., Chaudry, R. V., & Polivka, B. J. (2011). Regional differences as barriers to body mass index screening described by Ohio school nurses. Journal of School Health, 81(8), 437-448.

Steele, R. G., Wu, Y. P., Jensen, C. D., Pankey, S., Davis, A. M., & Aylward, B. S. (2011). School nurses' perceived barriers to discussing weight with children and their families: a qualitative approach. Journal of School Health, 81(3), 128-137.

Thompson, H. R., Linchey, J. K., & Madsen, K. A. (2015). Peer Reviewed: Critical Elements of a School Report to Parents on Body Mass Index. Preventing chronic disease, 12.

Thorstensson, S., Blomgren, C., Sundler, A. J., & Larsson, M. (2018). To break the weight gain—A qualitative study on the experience of school nurses working with overweight children in elementary school. Journal of clinical nursing, 27(1-2), e251-e258.

Turner, G. L., Owen, S., & Watson, P. M. (2016). Addressing childhood obesity at school entry: Qualitative experiences of school health professionals. Journal of Child Health Care, 20(3), 304-313.

Tyler, D. O., & Horner, S. D. (2008). Collaborating with low-income families and their overweight children to improve weight-related behaviors: An intervention process evaluation. Journal for Specialists in Pediatric Nursing, 13(4), 263-274.

Weatherson, K. A., McKay, R., Gainforth, H. L., & Jung, M. E. (2017). Barriers and facilitators to the implementation of a school-based physical activity policy in Canada: application of the theoretical domains framework. BMC public health, 17(1), 835.

Table of included study characteristics

Study	Research aims	Country	Study design	Sample (n)	Data collection	Data analysis
Bartelink 2019	To generate and share knowledge and experiences on how to implement changes in the complex school system to integrate school health promotion.	Netherlands	Mixed methods (process evaluation)	4 School coordinators, 4 PE coordinators, 4 Health promoters, 1 project leader (n=13)	Semi-structured interviews, observations, and analysis of minutes of meetings	Thematic analysis
Bergström 2015	To explore the views of teachers and parents regarding factors influencing the implementation of a school-based parental support programme to promote physical activity and healthy diet.	Sweden	Qualitative	3 Teachers, 14 parents (n=17)	Interviews, discussion groups	Content analysis
Bergström 2020	To explore barriers to and facilitators for the implementation of a parental programme to promote physical activity and healthy dietary habits in a school context.	Sweden	Qualitative	School nurses and principals (n=17)	Focus groups (nurses), interviews (principals)	Content analysis
Booth 2009	To examine the perceptions of parents of school-aged children regarding child and adolescent overweight and obesity	Australia	Qualitative	Parents (n=55)	Focus groups	Content analysis
Clarke 2017	To explore the views of headteachers, from a wide range of schools, on the role of primary schools in preventing obesity	UK	Qualitative	Headteachers (n=22)	Focus groups, interviews	Thematic analysis
Dariotis 2017	To improve the delivery of mindful yoga and other school-based programs by presenting perspectives from teachers and students	USA	Qualitative	22 Students, 9 teachers (31)	Focus groups, interviews	Thematic analysis
Ganter 2016	To capture the perspectives of these on-the-ground experts regarding major factors contributing to childhood obesity as well as gaps in current prevention and control efforts	USA	Qualitative	Stakeholders from: Schools (15), after school (8), early education (3), primary healthcare (7), and	Interviews	Grounded theory

				supplemental program for women and children (6) (n=39)		
Grimmett 2008	To compare parents' and children's reactions to a weighing and measurement program with weight feedback to parents in families with overweight or healthy-weight children	UK	Mixed methods	287 Parents, students, 358 (n=715)	Not stated	Not stated
Hall 2016	To explore how teachers describe their experience with nutrition education within the context of a phenomenology. What is common in their experiences.	USA	Qualitativ e	Teachers (n=10)	Semi-structured interviews, observation, teacher reflection	Thematic Analysis
Hart 2003	To investigate the existing psychosocial influences perceived by parents in relation to the eating and exercise behaviours of their primary school children	UK	Qualitativ e	Parents (n=41)	Focus group	Not stated
Howard-Drake 2016	To explore primary school headteachers' perspectives on childhood obesity and the perceived barriers and facilitators of prevention	UK	Qualitativ e	Headteacher s (n=14)	Semi-structured interviews	Thematic analysis
Jago 2015	To examine how the effectiveness of future primary (elementary) school diet and physical activity interventions could be improved.	UK	Qualitativ e	28 Teachers, 10 headteachers , 31 parents, 70 students (n=139)	Focus groups, interviews	Thematic analysis
Keough 2015	To determine how a required (BMI) screening and notification were viewed by caregivers and why they felt this way	USA	Qualitativ e	Parents (n=28)	Focus groups	Transcript based analysis
Kipping 2012	To identify possible methods to involve them in a school-based obesity prevention intervention, followed by a process evaluation of homework and school newsletters to involve parents	UK	Mixed methods	38 Parents, 32 students, 4 teachers (n=74)	Interviews, focus groups	Thematic analysis
Kubik 2007	To explore the opinions and beliefs of parents of elementary school students concerning school-based	USA	Qualitativ e	Parents (n=71)	Focus groups	Thematic analysis

	BMI screening programs, notification methods, message content, and health information needs related to promoting healthy weight for school-aged children					
Lloyd 2017	To ascertain the feasibility and acceptability of the design of the exploratory trial of the Healthy Lifestyles Programme (including the trial outcomes) and the HeLP Programme and whether it is able to engage schools, children and their families	UK	Qualitative	12 Teachers, 17 parents, 47 students (n=76)	Focus groups, interviews	Framework analysis
Luesse 2018	To investigate the challenges and facilitators to promoting a healthy environment at home and to identify communication preferences to inform intervention strategies for effectively reaching low-income urban minority families	USA	Qualitative	Parents (n=16)	Focus groups	Framework analysis
Mäenpää 2007	To describe the cooperation between sixth graders and the school nurses as experienced by the pupils; to obtain information for the development of holistic school health services and the school nurses' work.	Finland	Qualitative	Students (n=22)	Focus groups	Grounded theory
Magnusson 2012	To analyse school nurses' counselling of overweight and obese children in settings with many immigrants, focusing on content concerning food and physical activity and how this was communicated	Sweden	Qualitative	20 Students, 8 school nurses (n=28)	Audio-recording of school nurses	Content analysis
Moore 2010	To identify the eating behaviours of primary schoolchildren (aged 4–11 years) as displayed by pupils, and perceived by the school meal staff; to identify the feeding strategies implemented by school meal staff; and to identify the feeding outcomes sought by staff.	UK	Qualitative	Students, catering staff, supervisors (n=19, individual numbers unclear)	Observations, interviews	Not stated

Morrison-Sandberg 2011	To gain insight into current obesity related school nursing practice in elementary schools, the opinions and beliefs of school nurses and the local school community regarding school nurse-led obesity prevention programs, and interest among school nurses in implementing obesity prevention programs in the future	USA	Qualitative	School nurses (n=21)	Interviews	Content analysis
Moyer 2014	To assess educational material and BMI letter's readability/understandability and explore parents' responses to it; to explore specific potential barriers to effectiveness and parents' previous experiences with health care professionals discussing their child's weight	USA	Mixed methods	Parents/ caregivers (n=29)	Focus groups	Content anlaysis
Müllersdorf 2010	To describe how school nurses perceive their work with obese children with support of an action plan.	Sweden	Qualitative	School nurses (n=6)	Interviews	Content analysis
Norman 2016	To describe barriers and facilitators influencing implementation of the Healthy School Start (HSS) intervention in disadvantaged areas in Stockholm, Sweden, from the perspective of parents and teachers.	Sweden	Qualitative	10 Teachers, 14 parents (n=24)	Interviews	Content analysis
Passmore 2017	To explore students' and teachers' experiences of the Challenge Programme, and assess its impact on the students' physical activity levels.	Australia	Mixed methods	11 Teachers, 322 students (n=333)	Interviews, classroom discussion	Thematic analysis
Powell 2018a	To examine the descriptive comments of school nurses providing case management to students with severe obesity	USA	Mixed methods	37 students, school nurse *n* not stated (n=37)	Descriptive comments	Content analysis
Powell 2018b	To explore the experiences of school nurses providing care to children living with overweight and obesity; perceived barriers of successful intervention;	USA	Qualitative	School nurses (n=10)	Interviews	Content analysis

	how do nurses successfully implement interventions.					
Ramos 2015	To investigate elementary students' perceptions of Physical Education teacher credibility: to identify specific personal qualities, behaviours, and teaching skills that make PE teachers credible in the eyes of the students	USA	Qualitative	Students (n=8)	Open-ended questionnaire, student drawings, photograph elicitation, observations, group and individual interviews	Four-step flow model
Ruggieri 2016	To broaden our understanding of minority parents'/guardians' knowledge and perceptions of BMI measurements, screening programs, and report cards.	USA	Qualitative	Parents/ guardians (n=20)	Focus groups	Not stated
Schalkwijk 2015	To explore the expectations of obese children and their parents in relation to lifestyle interventions; to identify barriers to making lifestyle changes that parents and children face within their social context as well as the things that facilitate these; to identify the needs of obese children and their parents in the context of a lifestyle intervention	Netherlands	Qualitative	18 children, 24 parents (n=42)	Interviews	Thematic analysis
Schroeder 2017	To explore school nurses' perceived barriers to and facilitators of a school nurse-led obesity intervention to understand reasons for the low implementation rate (Healthy Options and Physical Activity Program – HOP)	USA	Qualitative	School nurses (n=19)	Interviews	Content analysis, heat maps
Schwartz 2015	To explore the perceptions of parents whose school-age children received a BMI referral letter stating their child is overweight	USA	Qualitative	Parents (n=21)	Interviews	Grounded theory
Stalter 2010	To identify facilitating factors and barriers of BMI screening practices among Ohio public elementary school nurses (policy factors, physical and social environment, school risk/protection factors,	USA	Qualitative	School nurses (n=25)	Focus groups	Content analysis

	access to quality health care factors					
Stalter 2011	To identify facilitating factors and barriers of BMI screening practices among Ohio public elementary school nurses (policy factors, physical and social environment, school risk/protection factors, access to quality health care factors) working in suburban, urban, and rural public elementary schools.	USA	Qualitative	School nurses (n=25)	Focus groups	Not stated
Steele 2011	To analyse school nurses' perceived barriers to addressing weight-related health issues with children and their families	USA	Qualitative	School nurses (n=22)	Focus groups	Not stated
Thompson 2015	To identify appropriate content for BMI reports (in preparation for a large randomized trial of school-based BMI screening and reporting.)	USA	Qualitative	Parents (n=79)	Focus groups	Thematic analysis
Thorstensson 2015	To describe the experiences of school nurses working with overweight schoolchildren.	Sweden	Qualitative	School nurses (n=6)	Interviews	RLR structure of whole–parts–whole described by Dahlberg et al.
Turner 2016	To explore the practice of school health professionals in addressing childhood obesity at school entry, with a view to explaining potential reasons for low referral rates and understanding how the role of school health professionals can be optimized to address childhood obesity at an early age	UK	Qualitative	3 Service managers, 16 school nurses, 7 child health practitioners (n=26)	Interviews, focus groups, open-ended questionnaires	Thematic analysis
Tyler 2008	To examine the collaborative negotiation process, an interventional approach being tested at a primary-care school-based clinic to help low-income families improve lifestyle and weight-related health indicators in their overweight children	USA	Qualitative	35 students and their parents, involved in recorded parent-child-provider interactions (n=111)	Structured field notes and audiotaping of sessions	Not stated

	(parent-child-provider interaction during intervention visits).					
Weatherson 2017	To understand teachers' barriers and facilitators to the implementation of the Daily Physical Activity (DPA) policy in one school district; to examine and compare barriers and facilitators according to how the teacher implemented the DPA policy during the instructional school day.	Canada	Qualitativ e	Teachers (n=13)	Interviews	Content analysis
