# Supplementary material for: Exploring primary school years interactions around child weight: A qualitative meta‐synthesis of school staff, parent, and child views and experiences
Source: Obes Rev. 2022 Apr 10;23(8):e13451. doi: 10.1111/obr.13451 (PMC9539573; doi:10.1111/obr.13451)
Supplement: Supplementary file 2 — Table S1. Template free‐text search terms and Boolean operators (subsequently adapted to specific database filters see below) [file OBR-23-e13451-s001.docx]

**Table 1.** Template free-text search terms and Boolean operators (subsequently adapted to specific database filters see below)

| **Boolean operators** |  | **AND** | **AND** | **AND** | **AND** |
| --- | --- | --- | --- | --- | --- |
| **Search terms** | stakeholder OR parent* OR mother OR father OR guardian OR teacher OR head teacher OR headteacher OR principal OR teaching assistant OR student OR pupil OR child* OR school nurs* OR governor OR famil* OR adolescent OR pediatric* OR paediatric* OR dietic* OR educat* OR nutritionist | communicat* OR conversation* OR discuss* OR talk* OR interaction* OR dialogue OR chat | weight OR obes* OR overweight OR growth OR diet OR physical activ* OR exercise* OR body mass | school* OR primary school OR infant school OR elementary school | Interview* OR focus group* OR qualitative |
|  |  |  |  |  |  |
|  |  |  |  |  |  |
|  |  |  |  |  |  |
|  |  |  |  |  |  |
|  |  |  |  |  |  |

**Ovid MEDLINE** (hits = 258, searched 25-04-19)

1. (stakeholder or parent* or mother or father or guardian or teacher or head teacher or principal or teaching assistant or student or pupil or child* or school* or nurse or governor or famil* or adolescent or pediatric* or paediatric* or dietic* or educat* or nutritionist).mp. [mp=title, abstract, original title, name of substance word, subject heading word, floating sub-heading word, keyword heading word, organism supplementary concept word, protocol supplementary concept word, rare disease supplementary concept word, unique identifier, synonyms]

2. limit 1 to (english language and full text and humans)

3. (communicat* or conversation* or discuss* or talk* or interaction* or dialogue or chat).mp. [mp=title, abstract, original title, name of substance word, subject heading word, floating sub-heading word, keyword heading word, organism supplementary concept word, protocol supplementary concept word, rare disease supplementary concept word, unique identifier, synonyms]

4. limit 3 to (english language and full text and humans)

5. (weight or obes* or overweight or growth or diet or physical activ* or exercise* or body mass).mp. [mp=title, abstract, original title, name of substance word, subject heading word, floating sub-heading word, keyword heading word, organism supplementary concept word, protocol supplementary concept word, rare disease supplementary concept word, unique identifier, synonyms]

6. limit 5 to (english language and full text and humans)

7. (school* or primary school or infant school or elementary school).mp. [mp=title, abstract, original title, name of substance word, subject heading word, floating sub-heading word, keyword heading word, organism supplementary concept word, protocol supplementary concept word, rare disease supplementary concept word, unique identifier, synonyms]

8. limit 7 to (english language and full text and humans)

9. (Interview* or focus group* or qualitative).mp. [mp=title, abstract, original title, name of substance word, subject heading word, floating sub-heading word, keyword heading word, organism supplementary concept word, protocol supplementary concept word, rare disease supplementary concept word, unique identifier, synonyms]

10. limit 9 to (english language and full text and humans)

11. 2 and 4 and 6 and 8 and 10

**EBSCOhost PsycINFO** (hits = 1,650, searched 19-06-19)

TX (stakeholder OR parent* OR mother OR father OR guardian OR teacher OR head teacher OR headteacher OR principal OR teaching assistant OR student OR pupil OR child* OR school nurs* OR governor OR famil* OR adolescent OR pediatric* OR paediatric* OR dietic* OR educat* OR nutritionist ) AND TX ( communicat* OR conversation* OR discuss* OR talk* OR interaction* OR dialogue OR chat ) AND TX ( weight OR obes* OR overweight OR growth OR diet OR physical activ* OR exercise* OR body mass ) AND TX ( school* OR primary school OR infant school OR elementary school) AND TX ( Interview* OR focus group* OR qualitative ). Search mode: Find all my search terms. Limits: full-text online; English language.

**EBSCOhost CINHAL Plus** (hits = 363, searched 24-06-19)

(stakeholder OR parent* OR mother OR father OR guardian OR teacher OR head teacher OR headteacher OR principal OR teaching assistant OR student OR pupil OR child* OR school nurs* OR governor OR famil* OR adolescent OR pediatric* OR paediatric* OR dietic* OR educat* OR nutritionist ) AND ( communicat* OR conversation* OR discuss* OR talk* OR interaction* OR dialogue OR chat ) AND ( weight OR obes* OR overweight OR growth OR diet OR physical activ* OR exercise* OR body mass ) AND ( school* OR primary school OR infant school OR elementary school ) AND ( Interview* OR focus group* OR qualitative ): Search mode Boolean/Phrase. Limits: English language, academic journals.

**EBSCOhost Education Research Complete** (hits = 1,655, searched 15-07-19)

(stakeholder OR parent* OR mother OR father OR guardian OR teacher OR head teacher OR headteacher OR principal OR teaching assistant OR student OR pupil OR child* OR school nurs* OR governor OR famil* OR adolescent OR pediatric* OR paediatric* OR dietic* OR educat* OR nutritionist ) AND ( communicat* OR conversation* OR discuss* OR talk* OR interaction* OR dialogue OR chat ) AND ( weight OR obes* OR overweight OR growth OR diet OR physical activ* OR exercise* OR body mass ) AND ( school* OR primary school OR infant school OR elementary school ) AND ( Interview* OR focus group* OR qualitative ): Search mode: Boolean/Phrase, Limits: English Language.
