# Supplementary material for: Exploring primary school years interactions around child weight: A qualitative meta‐synthesis of school staff, parent, and child views and experiences
Source: Obes Rev. 2022 Apr 10;23(8):e13451. doi: 10.1111/obr.13451 (PMC9539573; doi:10.1111/obr.13451)
Supplement: Supplementary file 3 — Data S2. Supporting Information [file OBR-23-e13451-s003.docx]

**References for studies included in review**

Bartelink, N., van Assema, P., Jansen, M., Savelberg, H., Moore, G., Hawkins, J., & Kremers, S. (2019). Process evaluation of the healthy primary School of the Future: the key learning points. BMC public health, 19(1), 698.

Bergström, H., Haggård, U., Norman, Å., Sundblom, E., Elinder, L. S., & Nyberg, G. (2015). Factors influencing the implementation of a school-based parental support programme to promote health-related behaviours—interviews with teachers and parents. BMC public health, 15(1), 541.

Bergström, H., Sundblom, E., Elinder, L. S., Norman, Å., & Nyberg, G. (2020). Managing Implementation of a Parental Support Programme for Obesity Prevention in the School Context: The Importance of Creating Commitment in an Overburdened Work Situation, a Qualitative Study. The Journal of Primary Prevention, 1-19.

Booth, M. L., King, L. A., Pagnini, D. L., Wilkenfeld, R. L., & Booth, S. L. (2009). Parents of school students on childhood overweight: the Weight of Opinion Study. Journal of paediatrics and child health, 45(4), 194-198.

Clarke, J., Pallan, M., Lancashire, E., & Adab, P. (2017). Obesity prevention in English primary schools: headteacher perspectives. Health promotion international, 32(3), 490-499.

Dariotis, J. K., Mirabal‐Beltran, R., Cluxton‐Keller, F., Feagans Gould, L., Greenberg, M. T., & Mendelson, T. (2017). A qualitative exploration of implementation factors in a school‐based mindfulness and yoga program: Lessons learned from students and teachers. Psychology in the Schools, 54(1), 53-69.

Ganter, C., Aftosmes-Tobio, A., Chuang, E., Blaine, R. E., Land, T., & Davison, K. K. (2016). Community stakeholders’ perceptions of major factors influencing childhood obesity, the feasibility of programs addressing childhood obesity, and persisting gaps. Journal of community health, 41(2), 305-314.

Grimmett, C., Croker, H., Carnell, S., & Wardle, J. (2008). Telling parents their child's weight status: psychological impact of a weight-screening program. Pediatrics, 122(3), e682-e688.

Hall, E., Chai, W., & Albrecht, J. A. (2016). A qualitative phenomenological exploration of teachers' experience with nutrition education. American Journal of Health Education, 47(3), 136-148.

Hart, K., Herriot, A., Bishop, J., & Truby, H. (2003). Promoting healthy diet and exercise patterns amongst primary school children: a qualitative investigation of parental perspectives. Journal of Human Nutrition and Dietetics, 16(2), 89-96.

Howard-Drake, E., & Halliday, V. (2016). Exploring primary school headteachers' perspectives on the barriers and facilitators of preventing childhood obesity. Journal of Public Health, 38(1), 44-52.

Jago, R., Rawlins, E., Kipping, R., Wells, S., Chittleborough, C., Peters, T., . . . Campbell, R. (2015). Lessons learned from the AFLY5 RCT process evaluation: implications for the design of physical activity and nutrition interventions in schools. BMC public health, 15(1), 1-10.

Keough, L. (2015). Caregivers of Underserved Minority Populations: Views and Opinions of the Role of Schools in BMI Screening, Education and Communication. Journal of Health Disparities Research & Practice, 8(3).

Kipping, R., Jago, R., & Lawlor, D. (2012). Developing parent involvement in a school-based child obesity prevention intervention: a qualitative study and process evaluation. Journal of Public Health, 34(2), 236-244.

Kubik, M. Y., Story, M., & Rieland, G. (2007). Developing school-based BMI screening and parent notification programs: findings from focus groups with parents of elementary school students. Health Education & Behavior, 34(4), 622-633.

Lloyd, J. J., & Wyatt, K. M. (2014). Qualitative findings from an exploratory trial of the Healthy Lifestyles Programme (HeLP) and their implications for the process evaluation in the definitive trial. BMC public health, 14(1), 578.

Luesse, H. B., Paul, R., Gray, H. L., Koch, P., Contento, I., & Marsick, V. (2018). Challenges and Facilitators to Promoting a Healthy Food Environment and Communicating Effectively with Parents to Improve Food Behaviors of School Children. Maternal and Child Health Journal, 22(7), 958-967. doi:10.1007/s10995-018-2472-7

Mäenpää, T., Paavilainen, E., & Åstedt-Kurki, P. (2007). Cooperation with school nurses described by Finnish sixth graders. International Journal of Nursing Practice, 13(5), 304-309. doi:10.1111/j.1440-172X.2007.00642.x

Magnusson, M. B., Kjellgren, K. I., & Winkvist, A. (2012). Enabling overweight children to improve their food and exercise habits – school nurses’ counselling in multilingual settings. Journal of clinical nursing, 21(17‐18), 2452-2460. doi:10.1111/j.1365-2702.2012.04113.x

Moore, S. N., Tapper, K., & Murphy, S. (2010). Feeding strategies used by primary school meal staff and their impact on children’s eating. Journal of Human Nutrition and Dietetics, 23(1), 78-84. doi:10.1111/j.1365-277X.2009.01009.x

Morrison-Sandberg, L. F., Kubik, M. Y., & Johnson, K. E. (2011). Obesity Prevention Practices of Elementary School Nurses in Minnesota:Findings From Interviews With Licensed School Nurses. The Journal of School Nursing, 27(1), 13-21. doi:10.1177/1059840510386380

Moyer, L. J., Carbone, E. T., Anliker, J. A., & Goff, S. L. (2014). The Massachusetts BMI letter: A qualitative study of responses from parents of obese children. Patient education and counseling, 94(2), 210-217. doi:https://doi.org/10.1016/j.pec.2013.10.016

Müllersdorf, M., Zuccato, L. M., Nimborg, J., & Eriksson, H. (2010). Maintaining professional confidence – monitoring work with obese schoolchildren with support of an action plan. Scandinavian Journal of Caring Sciences, 24(1), 131-138. doi:10.1111/j.1471-6712.2009.00696.x

Norman, Å., Nyberg, G., Elinder, L. S., & Berlin, A. (2016). One size does not fit all–qualitative process evaluation of the Healthy School Start parental support programme to prevent overweight and obesity among children in disadvantaged areas in Sweden. BMC public health, 16(1), 37. doi:10.1186/s12889-016-2701-1

Passmore, E., Donato-Hunt, C., Maher, L., Havrlant, R., Hennessey, K., Milat, A., & Farrell, L. (2017). Evaluation of a pilot school-based physical activity challenge for primary students. Health Promotion Journal of Australia, 28(2), 103-109.

Powell, S. B., Engelke, M. K., & Neil, J. A. (2018b). Seizing the moment: Experiences of school nurses caring for students with overweight and obesity. The Journal of School Nursing, 34(5), 380-389.

Powell, S. B., Engelke, M. K., & Swanson, M. S. (2018a). Quality of Life in School-Age Children with Obesity.

Ramos, N. C., & Mccullick, B. A. (2015). Elementary students’ construct of physical education teacher credibility. Journal of teaching in physical education, 34(4), 560-575.

Ruggieri, D. G., & Bass, S. B. (2016). African-American Parents' knowledge and perceptions about BMI measurements, school-based BMI screening programs, and BMI report cards: results from a qualitative investigation and implications for school-to-parent communication. Journal of racial and ethnic health disparities, 3(2), 320-330.

Schalkwijk, A., Bot, S., De Vries, L., Westerman, M., Nijpels, G., & Elders, P. (2015). Perspectives of obese children and their parents on lifestyle behavior change: a qualitative study. International Journal of Behavioral Nutrition and Physical Activity, 12(1), 1-8.

Schroeder, K., & Smaldone, A. (2017). What barriers and facilitators do school nurses experience when implementing an obesity intervention? The Journal of School Nursing, 33(6), 456-466.

Schwartz, M. (2015). Parental perceptions of body mass index notification: a qualitative study. Journal of School Health, 85(10), 714-721.

Stalter, A. M., Chaudry, R. V., & Polivka, B. J. (2010). Facilitating factors and barriers to BMI screening in schools. The Journal of School Nursing, 26(4), 320-330.

Stalter, A. M., Chaudry, R. V., & Polivka, B. J. (2011). Regional differences as barriers to body mass index screening described by Ohio school nurses. Journal of School Health, 81(8), 437-448.

Steele, R. G., Wu, Y. P., Jensen, C. D., Pankey, S., Davis, A. M., & Aylward, B. S. (2011). School nurses' perceived barriers to discussing weight with children and their families: a qualitative approach. Journal of School Health, 81(3), 128-137.

Thompson, H. R., Linchey, J. K., & Madsen, K. A. (2015). Peer Reviewed: Critical Elements of a School Report to Parents on Body Mass Index. Preventing chronic disease, 12.

Thorstensson, S., Blomgren, C., Sundler, A. J., & Larsson, M. (2018). To break the weight gain—A qualitative study on the experience of school nurses working with overweight children in elementary school. Journal of clinical nursing, 27(1-2), e251-e258.

Turner, G. L., Owen, S., & Watson, P. M. (2016). Addressing childhood obesity at school entry: Qualitative experiences of school health professionals. Journal of Child Health Care, 20(3), 304-313.

Tyler, D. O., & Horner, S. D. (2008). Collaborating with low‐income families and their overweight children to improve weight‐related behaviors: An intervention process evaluation. Journal for Specialists in Pediatric Nursing, 13(4), 263-274.

Weatherson, K. A., McKay, R., Gainforth, H. L., & Jung, M. E. (2017). Barriers and facilitators to the implementation of a school-based physical activity policy in Canada: application of the theoretical domains framework. BMC public health, 17(1), 835.

Table of included study characteristics

| **Study** | **Research aims** | **Country** | **Study design** | **Sample (n)** | **Data collection** | **Data analysis** |
| --- | --- | --- | --- | --- | --- | --- |
| Bartelink 2019 | To generate and share knowledge and experiences on how to implement changes in the complex school system to integrate school health promotion. | Netherlands | Mixed methods (process evaluation) | 4 School coordinators, 4 PE coordinators, 4 Health promoters, 1 project leader (n=13) | Semi-structured interviews, observations, and analysis of minutes of meetings | Thematic analysis |
| Bergström 2015 | To explore the views of teachers and parents regarding factors influencing the implementation of a school-based parental support programme to promote physical activity and healthy diet. | Sweden | Qualitative | 3 Teachers, 14 parents (n=17) | Interviews, discussion groups | Content analysis |
| Bergström 2020 | To explore barriers to and facilitators for the implementation of a parental programme to promote physical activity and healthy dietary habits in a school context. | Sweden | Qualitative | School nurses and principals (n=17) | Focus groups (nurses), interviews (principals) | Content analysis |
| Booth 2009 | To examine the perceptions of parents of school-aged children regarding child and adolescent overweight and obesity | Australia | Qualitative | Parents (n=55) | Focus groups | Content analysis |
| Clarke 2017 | To explore the views of headteachers, from a wide range of schools, on the role of primary schools in preventing obesity | UK | Qualitative | Headteachers (n=22) | Focus groups, interviews | Thematic analysis |
| Dariotis 2017 | To improve the delivery of mindful yoga and other school-based programs by presenting perspectives from teachers and students | USA | Qualitative | 22 Students, 9 teachers (31) | Focus groups, interviews | Thematic analysis |
| Ganter 2016 | To capture the perspectives of these on-the-ground experts regarding major factors contributing to childhood obesity as well as gaps in current prevention and control efforts | USA | Qualitative | Stakeholders from: Schools (15), after school (8), early education (3), primary healthcare (7), and supplemental program for women and children (6) (n=39) | Interviews | Grounded theory |
| Grimmett 2008 | To compare parents’ and children’s reactions to a weighing and measurement program with weight feedback to parents in families with overweight or healthy-weight children | UK | Mixed methods | 287 Parents, students, 358 (n=715) | Not stated | Not stated |
| Hall 2016 | To explore how teachers describe their experience with nutrition education within the context of a phenomenology. What is common in their experiences. | USA | Qualitative | Teachers (n=10) | Semi-structured interviews, observation, teacher reflection | Thematic Analysis |
| Hart 2003 | To investigate the existing psychosocial influences perceived by parents in relation to the eating and exercise behaviours of their primary school children | UK | Qualitative | Parents (n=41) | Focus group | Not stated |
| Howard-Drake 2016 | To explore primary school headteachers’ perspectives on childhood obesity and the perceived barriers and facilitators of prevention | UK | Qualitative | Headteachers (n=14) | Semi-structured interviews | Thematic analysis |
| Jago 2015 | To examine how the effectiveness of future primary (elementary) school diet and physical activity interventions could be improved. | UK | Qualitative | 28 Teachers, 10 headteachers, 31 parents, 70 students (n=139) | Focus groups, interviews | Thematic analysis |
| Keough 2015 | To determine how a required (BMI) screening and notification were viewed by caregivers and why they felt this way | USA | Qualitative | Parents (n=28) | Focus groups | Transcript based analysis |
| Kipping 2012 | To identify possible methods to involve them in a school-based obesity prevention intervention, followed by a process evaluation of homework and school newsletters to involve parents | UK | Mixed methods | 38 Parents, 32 students, 4 teachers (n=74) | Interviews, focus groups | Thematic analysis |
| Kubik 2007 | To explore the opinions and beliefs of parents of elementary school students concerning school-based BMI screening programs, notification methods, message content, and health information needs related to promoting healthy weight for school-aged children | USA | Qualitative | Parents (n=71) | Focus groups | Thematic analysis |
| Lloyd 2017 | To ascertain the feasibility and acceptability of the design of the exploratory trial of the Healthy Lifestyles Programme (including the trial outcomes) and the HeLP Programme and whether it is able to engage schools, children and their families | UK | Qualitative | 12 Teachers, 17 parents, 47 students (n=76) | Focus groups, interviews | Framework analysis |
| Luesse 2018 | To investigate the challenges and facilitators to promoting a healthy environment at home and to identify communication preferences to inform intervention strategies for effectively reaching low-income urban minority families | USA | Qualitative | Parents (n=16) | Focus groups | Framework analysis |
| Mäenpää 2007 | To describe the cooperation between sixth graders and the school nurses as experienced by the pupils; to obtain information for the development of holistic school health services and the school nurses’ work. | Finland | Qualitative | Students (n=22) | Focus groups | Grounded theory |
| Magnusson 2012 | To analyse school nurses’ counselling of overweight and obese children in settings with many immigrants, focusing on content concerning food and physical activity and how this was communicated | Sweden | Qualitative | 20 Students, 8 school nurses (n=28) | Audio-recording of school nurses | Content analysis |
| Moore 2010 | To identify the eating behaviours of primary schoolchildren (aged 4–11 years) as displayed by pupils, and perceived by the school meal staff; to identify the feeding strategies implemented by school meal staff; and to identify the feeding outcomes sought by staff. | UK | Qualitative | Students, catering staff, supervisors (n=19, individual numbers unclear) | Observations, interviews | Not stated |
| Morrison-Sandberg 2011 | To gain insight into current obesity related school nursing practice in elementary schools, the opinions and beliefs of school nurses and the local school community regarding school nurse-led obesity prevention programs, and interest among school nurses in implementing obesity prevention programs in the future | USA | Qualitative | School nurses (n=21) | Interviews | Content analysis |
| Moyer 2014 | To assess educational material and BMI letter’s readability/understandability and explore parents’ responses to it; to explore specific potential barriers to effectiveness and parents' previous experiences with health care professionals discussing their child's weight | USA | Mixed methods | Parents/ caregivers (n=29) | Focus groups | Content anlaysis |
| Müllersdorf 2010 | To describe how school nurses perceive their work with obese children with support of an action plan. | Sweden | Qualitative | School nurses (n=6) | Interviews | Content analysis |
| Norman 2016 | To describe barriers and facilitators influencing implementation of the Healthy School Start (HSS) intervention in disadvantaged areas in Stockholm, Sweden, from the perspective of parents and teachers. | Sweden | Qualitative | 10 Teachers, 14 parents (n=24) | Interviews | Content analysis |
| Passmore 2017 | To explore students’ and teachers’ experiences of the Challenge Programme, and assess its impact on the students’ physical activity levels. | Australia | Mixed methods | 11 Teachers, 322 students (n=333) | Interviews, classroom discussion | Thematic analysis |
| Powell 2018a | To examine the descriptive comments of school nurses providing case management to students with severe obesity | USA | Mixed methods | 37 students, school nurse *n* not stated (n=37) | Descriptive comments | Content analysis |
| Powell 2018b | To explore the experiences of school nurses providing care to children living with overweight and obesity; perceived barriers of successful intervention; how do nurses successfully implement interventions. | USA | Qualitative | School nurses (n=10) | Interviews | Content analysis |
| Ramos 2015 | To investigate elementary students’ perceptions of Physical Education teacher credibility: to identify specific personal qualities, behaviours, and teaching skills that make PE teachers credible in the eyes of the students | USA | Qualitative | Students (n=8) | Open-ended questionnaire, student drawings, photograph elicitation, observations, group and individual interviews | Four-step flow model |
| Ruggieri 2016 | To broaden our understanding of minority parents'/guardians' knowledge and perceptions of BMI measurements, screening programs, and report cards. | USA | Qualitative | Parents/ guardians (n=20) | Focus groups | Not stated |
| Schalkwijk 2015 | To explore the expectations of obese children and their parents in relation to lifestyle interventions; to identify barriers to making lifestyle changes that parents and children face within their social context as well as the things that facilitate these; to identify the needs of obese children and their parents in the context of a lifestyle intervention | Netherlands | Qualitative | 18 children, 24 parents (n=42) | Interviews | Thematic analysis |
| Schroeder 2017 | To explore school nurses’ perceived barriers to and facilitators of a school nurse-led obesity intervention to understand reasons for the low implementation rate (Healthy Options and Physical Activity Program – HOP) | USA | Qualitative | School nurses (n=19) | Interviews | Content analysis, heat maps |
| Schwartz 2015 | To explore the perceptions of parents whose school-age children received a BMI referral letter stating their child is overweight | USA | Qualitative | Parents (n=21) | Interviews | Grounded theory |
| Stalter 2010 | To identify facilitating factors and barriers of BMI screening practices among Ohio public elementary school nurses (policy factors, physical and social environment, school risk/protection factors, access to quality health care factors | USA | Qualitative | School nurses (n=25) | Focus groups | Content analysis |
| Stalter 2011 | To identify facilitating factors and barriers of BMI screening practices among Ohio public elementary school nurses (policy factors, physical and social environment, school risk/protection factors, access to quality health care factors) working in suburban, urban, and rural public elementary schools. | USA | Qualitative | School nurses (n=25) | Focus groups | Not stated |
| Steele 2011 | To analyse school nurses’ perceived barriers to addressing weight-related health issues with children and their families | USA | Qualitative | School nurses (n=22) | Focus groups | Not stated |
| Thompson  2015 | To identify appropriate content for BMI reports (in preparation for a large randomized trial of school-based BMI screening and reporting.) | USA | Qualitative | Parents (n=79) | Focus groups | Thematic analysis |
| Thorstensson 2015 | To describe the experiences of school nurses working with overweight schoolchildren. | Sweden | Qualitative | School nurses (n=6) | Interviews | RLR structure of whole– parts–whole described by Dahlberg et al. |
| Turner 2016 | To explore the practice of school health professionals in addressing childhood obesity at school entry, with a view to explaining potential reasons for low referral rates and understanding how the role of school health professionals can be optimized to address childhood obesity at an early age | UK | Qualitative | 3 Service managers, 16 school nurses, 7 child health practitioners (n=26) | Interviews, focus groups, open-ended questionnaires | Thematic analysis |
| Tyler 2008 | To examine the collaborative negotiation process, an interventional approach being tested at a primary-care school-based clinic to help low-income families improve lifestyle and weight-related health indicators in their overweight children (parent-child-provider interaction during intervention visits). | USA | Qualitative | 35 students and their parents, involved in recorded parent-child-provider interactions (n=111) | Structured field notes and audiotaping of sessions | Not stated |
| Weatherson 2017 | To understand teachers’ barriers and facilitators to the implementation of the Daily Physical Activity (DPA) policy in one school district; to examine and compare barriers and facilitators according to how the teacher implemented the DPA policy during the instructional school day. | Canada | Qualitative | Teachers (n=13) | Interviews | Content analysis |
|  |  |  |  |  |  |  |
